# Alternative Splicing of *MoPTEN* Is Important for Growth and Pathogenesis in *Magnaporthe oryzae*

**DOI:** 10.3389/fmicb.2021.715773

**Published:** 2021-07-16

**Authors:** Shaowei Wang, Hao Liang, Yi Wei, Penghui Zhang, Yuejia Dang, Guihua Li, Shi-Hong Zhang

**Affiliations:** ^1^College of Plant Sciences, Jilin University, Changchun, China; ^2^Center for Extreme-Environmental Microorganisms, Shenyang Agricultural University, Shenyang, China; ^3^College of Plant Protection, Shenyang Agricultural University, Shenyang, China

**Keywords:** alternative splicing, *MoPTEN*, growth, pathogenesis, *Magnaporthe oryzae*

## Abstract

Human PTEN, a dual-phosphatase tumor suppressor, is frequently dysregulated by alternative splicing. Fungi harbor *PTEN* homologs, but alternative splicing of fungal *PTENs* has not been reported as far as we know. Here, we described an alternative splicing case in the *PTEN* homolog of *Magnaporthe oryzae* (*MoPTEN*). Two splice variants of *MoPTEN* were detected and identified, which are resulted from an intron retention and exclusion (*MoPTEN-1/2*). Both proteins were different in lipid and protein phosphatase activity and in expression patterns. The *MoPTEN* deletion mutant (Δ*MoPTEN*) showed the defects in conidiation, appressorium formation, and pathogenesis. Δ*MoPTEN* could be completely restored by *MoPTEN*, but rescued partially by *MoPTEN-1* in the defect of conidium and appressorium formation, and by *MoPTEN-2* in the defect of invasive development. Assays to assess sensitivity to oxidative stress reveal the involvement of *MoPTEN-2* in scavenging exogenous and host-derived H_2_O_2_. Taken together, *MoPTEN* undergoes alternative splicing, and both variants cooperatively contribute to conidium and appressorium development, and invasive hyphae growth in plant cells, revealing a novel disease development pathway in *M. oryzae*.

## Introduction

Protein phosphorylation, co-regulated by kinase and phosphatase, plays multiple important roles in eukaryotic organisms (Zolnierowicz and Bollen, 2000; Bauman and Scott, 2002). Kinase catalyzes the transfer of phosphate from a donor to a protein resulting in phosphorylated proteins; conversely, phosphatase hydrolyses phosphorylated proteins leading to the removal of phosphate (dephosphorylation). Based on the homology, structure, and substrates, protein phosphatases are divided into two families: serine/threonine protein phosphatases and protein tyrosine phosphatases (PTPs) (Pao et al., 2007; Shi, 2009). Protein tyrosine phosphatase family is very complicated, and it can be subdivided into several classes, such as specific protein-tyrosine phosphatases and dual-specificity phosphatases (Andersen et al., 2001; Moorhead et al., 2007; Pao et al., 2007).

The human tumor suppressor, PTEN/MMAC1/TEP1 (PTEN, phosphatase deleted on chromosome ten; MMAC1, mutated in multiple advanced cancers; TEP1, tensin-like phosphatase) is a dual-specificity phosphatase based on the conserved catalytic domain (Li et al., 1997a; Steck et al., 1997). As a protein phosphatase, PTEN dephosphorylates the corresponding phosphorylated proteins (Shi et al., 2014; Shinde and Maddika, 2016). However, different from the classic dual-specificity phosphatases, PTEN catalyzes the dephosphorylation of both protein and non-protein substrates and preferentially dephosphorylates the secondary messenger phosphatidylinositol-3,4,5-trisphosphate (PIP3) to phosphatidylinositol-4,5-diphosphate (PIP2), which is essential for regulating the highly oncogenic pro-survival phosphatidylinositol-3-phosphate kinase (PI3K) signaling pathway (Lee et al., 1999; Salmena et al., 2008).

PTEN protein is encoded by a unique gene (Liang et al., 2014). However, alternative splicing increases the number of transcripts. More splice variants, by intron retention or exon exclusion, have been detected to be inactive forms of PTEN (Sharrard and Maitland, 2000; Agrawal and Eng, 2006). PTEN plays multiple important roles in many cellular activities. In addition to tumor suppression, it is also involved in metabolism, tissue homeostasis, differentiation, and neurological diseases (Ramaswamy et al., 1999; Rademacher and Eickholt, 2019). PTEN and homologs are evolutionarily conserved in humans and mammals, but a small number of PTEN homologs have been found in fungi. *TEP1* (YNL128w), the first fungal *PTEN* gene, was cloned in the budding yeast *Saccharomyces cerevisiae* (Li et al., 1997b). The yeast PTEN homolog, like the mammalian counterpart, functions in the phosphatidylinositol pathway; but differently, the *PTEN*/*TEP1* is required for yeast sporulation (Heymont et al., 2000). In filamentous fungal pathogens, homologs of *TEP1/PTEN* appear to be associated with conidium formation and pathogenesis (Zhang et al., 2010; Vijayakrishnapillai et al., 2018; Wang et al., 2021).

*Magnaporthe oryzae* is the causal agent of rice blast worldwide. Host cell invasion is initiated by developed conidia, which occurs outside plant cells and involves conidium germination, tube elongation, appressorium maturation and differentiation (Howard et al., 1991; Talbot, 2003). After penetration, successful development of invasive hyphae determines the severity of blast (Heath et al., 1990; Kankanala et al., 2007). In a whole cycle of disease development, multiple phosphorylation dependent signaling pathways are required, which include the Pmk1 mitogen-activated protein (MAP) kinase, cyclic AMP dependent protein kinase A and Pkc1-Mps1 MAP kinase pathways (Li et al., 2012). Considering the balance of phosphorylation, dephosphorylation with different phosphatases are inevitably activated (Franck et al., 2015). So far, serine/threonine protein phosphatase PP2Ac (Du et al., 2013) and MoPpe1 (Qian et al., 2018) were demonstrated to play vital roles in the pathogenic development of *M. oryzae*. Recently, Sun et al. (2018) reported in the rice blast fungus that Target-of-Rapamycin (TOR) nutrient-signaling pathway plays an important role in mediating plant-fungal biotrophic interface membrane integrity and inhibiting the formation of appressorium in *M. oryzae* by suppressing the downstream of the cPKA pathway (Marroquin-Guzman and Wilson, 2015). Importantly, a dual-specificity phosphatase MoYvh1 was shown to play important roles in scavenging of host-derived reactive oxygen species (ROS) and subverting rice defense in *M. oryzae* (Liu et al., 2018).

Despite the importance of TEP1/PTEN in fungi, there are only a few reports on the function and regulation of its homologs in the filamentous fungi. In this research, we biologically analyzed the PTEN-like gene through creating several mutant strains in the rice blast fungus *M. oryzae*. Biological and molecular data reveal that an alternative splicing of *MoPTEN* is important for growth, development, and pathogenesis in *M. oryzae*.

## Materials and Methods

### Fungal Strains and Growth Conditions

The wild-type strain *M. oryzae* JL0910 was previously isolated and purified from the rice cultivar Jijing88, which is widely planted in Jilin Province, China (Li et al., 2015). All strains, including the four strains generated in this study, were cultured on complete media (CM) agar plates (1 g/L yeast extract, 0.5 g/L enzyme hydrolyzed casein, 0.5 g/L acid hydrolyzed casein, 10 g/L glucose, 1 g/L Ca(NO_3_)_2_⋅4H_2_O, 0.2 g/L KH_2_PO_4_, 0.25 g/L MgSO_4_⋅7H_2_O, 0.15 g/L NaCl, and 15 g/L agar), or potato dextrose agar (PDA) (200 g/L peeled potatoes, 20 g/L glucose, and 15 g/L agar), and stored on filter paper at −20°C. The strains were cultured for 7 days on CM for assessment of their growth rates. Each test was repeated at least three times. Mycelia used for nucleic acid extraction were prepared by growing the relevant strains in 100 mL liquid CM for 3 days at 25°C with gentle rocking at 150 rpm under bright light. For sporulation analysis and conidia harvesting, the strains were inoculated on oatmeal-tomato agar medium (OTA) and incubated at 25°C for 10 days in the dark (Cui et al., 2015). After the aerial hyphae of the colonies had been washed away using sterilized distilled water, the strains were continually grown for 3 days under a fluorescent light.

*Saccharomyces cerevisiae* BY4743 and the *TEP1/PTEN* deletion mutant strains (Invitrogen, Beijing, China) were used for functional complementation test. The PI3K inhibitor wortmannin (KY12420, Sigma, Beijing, China) was prepared in dimethylsulfoxide (DMSO) as stock solution (25 mg/mL) and stored at −20°C. The yeast *S. cerevisiae* transformation was performed by the lithium acetate procedure. For yeast gene expression, YPB-ADHpt promoter and terminator regions of ADH1 gene in YPB1 was used (Bertram et al., 1996). All yeast strains were cultured according to Li et al. (2018).

### DNA, RNA, and Protein Manipulations

Total RNA or DNA were extracted using RNA or DNA extraction kits (Sangon Biotech, Shanghai, China). First-strand cDNA was synthesized from 2.0 μg of total RNA using Avian Myeloblastosis Virus reverse transcriptase (Promega, Madison, WI, United States). The cDNA samples were 10-fold diluted and used as templates for RT-PCR.

PCR DIG Probe Synthesis Kit (Sigma-Aldrich, Shanghai, China) was used for Southern blot. The fragment with the hygromycin B phosphotransferase gene (*HPH*) was used as a hybridization probe, and the *HPH* probe fragment was amplified by using primer pair HPH1 and HPH2 (Supplementary Table 1). The genomic DNA of S28515 was digested with *Eco*RV and *Xba*I, respectively.

The flanking sequence of T-DNA insertion locus of S28515 was isolated by Thermal asymmetric interlaced PCR (Tail-PCR) (Liu and Whittier, 1995), and amplified by arbitrary degenerate primers (AD4) and T-DNA specific primers (RB1-3). The PCR used high-fidelity polymerase (TaKaRa, Dalian, China) for amplification. All the PCR fragments were sequenced (Sangon Biotech, Shanghai, China) and analyzed through NCBI BLAST^1^. Transcript analysis of the *MoPTEN* gene was performed based on semi-quantitative RT-PCR (semi-qRT-PCR). RNA samples were extracted from the vegetative mycelia, conidia, germinated conidia, appressoria at different developmental stages, and diseased leaves at certain hours post-inoculation (hpi), respectively. Total RNA was used to synthesize the first-strand cDNA. The *MoACTIN* gene (MGG_03982) was used as an endogenous control. The primers semi-YP-F/R were used for the *MoPTEN* gene, and the primers semi-actin-F/R were used for the actin gene. Agarose gel electrophoresis (2%) was used to display the *MoPTEN* expression level and pattern. The experiments were independently repeated twice with three biological replicates, and all primer pairs used herein are listed in Supplementary Table 1.

For the recombinant protein preparation, the full-length fragments of *MoPTEN-1* (1,974 bp) and *MoPTEN-2* (1,890 bp) were amplified using PCR with a pair of primers (MBD-F/R) containing *Eco*RI and *Hind*III restriction sites. The PCR product was subcloned into the pMD-18T vector (TaKaRa, Dalian, China), and the fragment containing the *MoPTEN-1* or *MoPTEN-2* gene ORF was cloned in-frame into the pET-28a (+) vector (Novagen, Shanghai, China). Using the same strategy, other expression vectors harboring the gene fragments corresponding to either *MoPTEN-1* or *MoPTEN-2* were generated using the primers listed in Supplementary Table 1. *Escherichia coli* strains BL21 and DH5α were used as hosts for the plasmid DNA amplification and protein expression, and *E. coli* cells harboring the pET-28a*::MoPTEN-1* or *MoPTEN-2* plasmid were grown in LB medium at 30°C. Once the OD_600_ reached 0.6, IPTG (isopropylthio-β-D-galactoside) was added to a final concentration of 1 mM, and the cells were cultured for another 5–6 h. Recombinant protein was purified from *E. coli* cells using a Ni^2+^-NTA purification kit according to the manufacturer’s instructions (Novagen, Shanghai, China).

### Lipid-/Protein-Phosphatase Activity Assays

The lipid-/protein-phosphatase activity of the recombinant MoPTEN-1 and MoPTEN-2 proteins were assayed on a soluble PIP3 or a synthetic phosphopeptide (DADEpYLIPQQG) by using a malachite green- or a molybdenum blue-reaction colorimetry phosphatase assay system (GENMED Scientifics, Shanghai, China) according to the manufacturer’s instructions. The substrates were diluted to series of concentrations in phosphate free buffer (25 mM Tris, 100 mM NaCl pH 7.6). All reactions were incubated at 37°C for 30 min and terminated by adding 2 μL NaOH (10 M). A 50 μL of malachite green or molybdenum blue (BIOMOL Enzo Life Sciences, Shenzhen, China) were used to detect the released phosphate. Absorption at 620 or 660 nm was quantified in a NanoPhotometer-N50 microplate spectrophotometer (München, Germany). Phosphate standards were utilized to quantify the phosphate released by each sample. Samples were run in triplicate and results were normalized to a water control. Recombinant human PTEN (BioVision4838-5, San Francisco, CA, United States) or human PTP1B (P6244, Sigma-Aldrich, United States) was used as positive control.

### Generation of the *GFP::PTEN* Gene, *PTEN* Gene Deletion and Complementation Strains

The vector pCAMBIA1303 containing promoter-MoPTEN-GFP fusion gene was constructed (Supplementary Figure 1). Promoter-MoPTEN (3,107 bp) was amplified using primers Prom-PTEN-F/R, then ligated into the pCAMBIA1303 (*Spe* I restricted), generating pCAMBIA1303-MoPTEN-GFP. The *Agrobacterium tumefaciens* mediated transformation (ATMT) protocol was conducted according to Khan et al. (2014). Transformants were screened on PDA plates with 200 μg/mL HygB. The GFP tag was amplified with the GFP-F/R primers for identification. The fusion gene expression was amplified with primers Mq-YP-F/R. The *MoACTIN* gene (MGG_03982) was amplified with the primers Mq-actin-F/R to serve as an endogenous reference.

To generate the *MoPTEN* deletion strain Δ*MoPTEN*, the *MoPTEN* gene was replaced by the hygromycin resistant cassette (*HPH*). To construct the replacement vector, the flanking sequences were amplified with their corresponding primer pairs (MGG-qc-LF/LR and MGG-qc-RF/RR), treated with restriction enzymes and ligated into the *Eco*RI – *Kpn*I and *Xba*I – *Sal*I sites of the pXEH 2.0 vector to construct a knockout vector (Supplementary Figures 2A,B). The three pairs of specific primers MoPTEN-F/R, HYG-YZ-F/R, and M-L-F/R were used to detect the genomes of transformants. The expression level of the subcellular deletion strains was detected using qRT-PCR (Supplementary Figure 2C).

Three complementation strains of Δ*MoPTEN* were constructed by using the full-length DNA sequence of the *MoPTEN* gene (2,114 bp), the *MoPTEN-1* (1,974 bp) cDNA containing two synonymous mutation sites at T^981^A and A^1062^T, and the *MoPTEN-2* (1,890 bp) cDNA (second intron spliced form). These three fragments were, respectively, amplified by using the primers MGG-hb-F/R, and ligated into the *Sma*I restricted site of the pKD7-Red vector (G418-resistance) (a kind gift from Dr. Hongkai Wang and Dr. Jianping Lu, Zhejiang University, China) (Supplementary Figures 3A,B). The recombinant vectors were introduced into the Δ*MoPTEN* strains using *A. tumefaciens* mediated transformation method. Complementation transformants were screened on PDA agar plates supplemented with 300 μg/mL G418 and were confirmed using PCR and semi-qRT-PCR (Supplementary Figures 3C,D). The synonymous mutation was performed using TaKaRa MutanBEST Kit (TaKaRa, Japan, Dalian), a pair of primers MoPTEN-1(SM)-F/R with nucleotide substitution were showed in Supplementary Table 1.

### Conidiation Quantification, Appressorium Induction, and Inoculation

After 10 days of cultivation on OTA, conidia were collected with 5 mL of distilled water, filtered through three layers of lens paper (Sealee, Japan), and counted with a hemacytometer under a Nikon Eclipse Ni-U microscope (Nikon, Tokyo, Japan). Conidial germination and appressorium formation were measured on a hydrophobic surface (plastic cover slips or gel-bond films) and onion epidermal cells. Conidial suspensions of 30 μL (1 × 10^5^ conidia/mL) were dropped onto a hydrophobic surface or onion epidermal cells and were placed in a moistened box at 25°C. Appressorium formation rate was then calculated under the microscope at 12 hpi while photographs were taken at 24 hpi. More than 100 appressoria were counted for each strain and the experiment was repeated three times.

For the leaf drop-inoculation assay, 10 μL of a conidial suspension (1 × 10^5^ conidia/mL) was dropped onto 10-cm leaf fragments cut from 2-week-old rice seedlings. Leaves pre-abraded with blade or unwounded were used for drop-inoculation. The inoculated leaves were placed on plastic plates with 90% humidity at 25°C for 24 h in the dark, then incubated in a 12-h light/12-h dark cycle until the large lesions appeared. For the inoculation of intact rice leaves, a conidial suspension (1 × 10^5^ conidia/mL) was sprayed onto the leaves using an air sprayer. The inoculated plants were placed in a high humidity of 90% chamber at 25°C for 24 h in the dark, then transferred to a growth chamber with a 16-h light/8-h dark photoperiod. The different types of lesions on the 4 cm^2^ leaves were counted and photographed at 7–9 days post-inoculation (dpi). These experiments were performed in triplicate and repeated three times for each strain.

For microscopic observation of cuticle penetration and invasive hyphae growth, leaf sheaths and inoculation were prepared as described in Koga et al. (2004), and inoculated with 300 μL of conidial suspension (1 × 10^5^ conidia/mL) on the inner leaf sheath cuticle cells. After 48 h incubation under the 90% humid conditions at room temperature, the leaf sheaths were observed under a microscope.

### H_2_O_2_ Stress Test, DAB Staining and DPI Treatment Assay

The wild type, mutant and complementation strains were continuously cultured on CM plates with concentrations of 2.5 and 5 mM H_2_O_2_ in the dark for 7 days at 25°C, and the fungal colonies were observed. The CM medium supplemented without H_2_O_2_ (0 mM) served as the control.

The 3,3′-diaminobenzidine (DAB) and diphenyleneiodonium (DPI) were used to detect the accumulation of H_2_O_2_ in plant cells infected by the different strains of *M. oryzae*. Rice leaf sheaths were injected with a conidial suspension at a concentration of 1 × 10^5^ conidia/mL. For DAB staining assay, the infected leaf sheaths were sliced after 48 hpi, and stained in DAB dye solution (1 mg/mL; pH 3.8) for 8 h and destained with ethanol/acetic acid (94:4, v/v) for 1 h (Marroquin-Guzman et al., 2017), and then observed under a microscope. For DPI treatment test, the conidia of wild type, mutant and complementation strains were treated with a concentration of 0.4 μM DPI, which will inhibit plant NADPH oxidase (NOX) but not to affect fungal physiology (Fernandez et al., 2014), before infecting leaf sheaths. At 48 hpi, the infected rice leaves were observed under a microscope. These experiments were performed in triplicate and repeated three times for each strain.

### Determination of Appressorial Melanin

Germinated conidia with or without appressoria were collected as samples to measure appressorial melanin, respectively. A 50 mg dried sample pellet was suspended in 6 mL NaOH solution (1 M) and continually heated at 121°C for 20 min. With 1 M NaOH as a blank control, the absorbance was measured at 405 nm with an ultraviolet spectrophotometer (Bio-Rad SmartSpec Plus, CA, United States) (Suryanarayanan et al., 2004). These experiments were performed in triplicate and repeated three times for each strain.

### Statistical Analysis

All quantitative data provided in this study represent the results of triplicate experiments independently performed at least three times. Origin 7.0 software (OriginLab Corp., Northampton, MA, United States) was used to analyze the data and determine the mean ± SD of enzyme activity, conidiation, rate of conidial germination, rate of appressorial formation, colony diameters, relative expression and different types of disease. The significance of the data was assessed using the Student’s *t*-test. *P* < 0.05 was considered statistically significant. Error bars represent the standard deviation.

## Results

### Identification of the H_2_O_2_ Sensitive and Virulence Defective Strain S28515

During host-pathogen interactions, the increased ROS in rice cells is a threat to *M. oryzae*. Accumulation of ROS such as H_2_O_2_ is known to govern the pathogen and host interaction. Most mutants with pathogenicity defect are impaired in antioxidation (Ding et al., 2010; Marroquin-Guzman et al., 2017; Liu et al., 2018; Li et al., 2020), thus we planned to obtain non-pathogenic mutants based on H_2_O_2_ primary screening.

**FIGURE 1:**
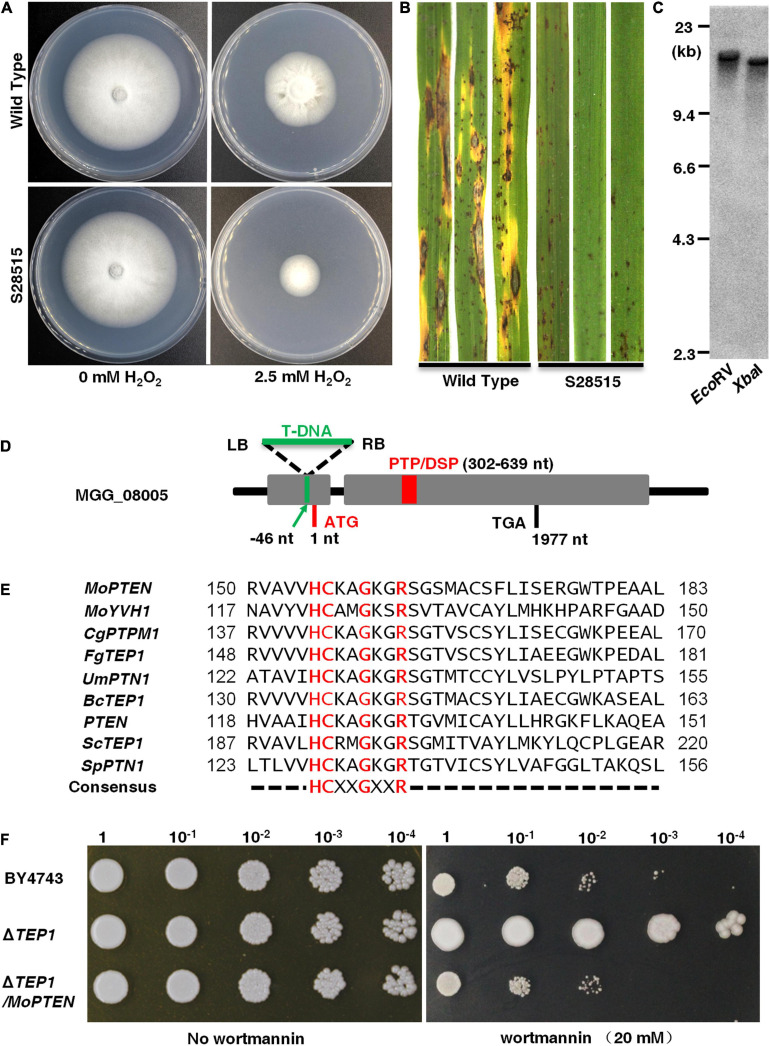
Identification of a screened H_2_O_2_ sensitive mutant S28515. **(A)** S28515 is more sensitive to 2.5 mM H_2_O_2_. The wild type and S28515 strains were cultured on complete media (CM) plates or CM containing H_2_O_2_ (2.5 mM) plates in the dark for 10 days at 25°C and then representative colonies were photographed 7 days post-inoculation (dpi). **(B)** S28515 is defective in pathogenicity. Conidial suspensions of the wild type and S28515 strains were sprayed on rice seedlings. Diseased leaves were photographed 7 dpi. **(C)** Southern blot analysis of S28515. The total genomic DNA was digested with *Eco*RV and *Xba*I, and probed with the partial *HPH* fragment. **(D)** Insertion site analysis. The green arrow indicates the MGG_08005 T-DNA insertion site. The gray area represents the mRNA transcriptional region of the gene MGG_08005. The red rectangle represents PTP/DSP domain. The capital letters ATG and TGA represent the translation start and stop sites, respectively. **(E)** Sequence alignment. The catalytic domain of *MoPTEN* was compared with the phosphatase catalytic domains of reported species, which include *Magnaporthe oryzae* Guy11, *Colletotrichum graminicola* M1.001, *Fusarium graminearum* PH-1, *Ustilago maydis* 521, *Botrytis cinerea* B05.10, *Homo sapiens*, *Saccharomyces cerevisiae* S288C, and *Schizosaccharomyces pombe*. **(F)** Functional complementation of *MoPTEN* for *ScTEP1* in *S. cerevisiae*. A 10 μL droplets containing the indicated concentration of yeast cells were inoculated on to the solid YPD medium plates (20 mM wortmannin added). Wortmannin resistant phenotype of Δ*TEP1* was restored to sensitivity by transferring the *MoPTEN* gene in the yeast. Representative plates were photographed 3 dpi.

From an *A. tumefaciens* mediated T-DNA insertion library, we screened a mutant S28515 that was sensitive to 2.5 mM H_2_O_2_ on CM plate (Figure 1A). In addition, the S28515 mutant failed to cause typical blast lesions and instead caused some pinhead-sized brown specks and restricted disease lesions (Figure 1B). Southern blot analysis indicated that a single-copy T-DNA fragment was inserted in the genome of S28515 (Figure 1C), confirming the phenotype is caused by the single T-DNA insertion.

Tail-PCR analysis was carried out to clone the disrupted locus. According to the bio-information of flanking sequences of the T-DNA insertion, the insertion site was determined to be 46 bp upstream of the start codon in MGG_08005 fragment (Figure 1D). The MGG_08005 locus, located on chromosome II of *M. oryzae*, has one intron and transcribes a length of 2,955 bp mRNA^2^. The open reading frame (ORF, 1,977 bp) encodes a protein of 658 amino acid residues with a predicted molecular mass of 72.6 kDa, which contains the consensus active site motif “HCXXGXXR” in most protein tyrosine phosphatases (Figure 1E; Supplementary Figure 4A).

Phylogenetic analysis showed that MGG_08005 protein and several selected homologs of PTEN/TEP1 were contained in the same clade (Supplementary Figure 4B), sharing 54% identity and 65% similarity with CgPTPM1, and 55% identity and 66% similarity with FgTEP1, but only 27% identity and 40% similarity with ScTep1, and 31% identity and 49% similarity with mammalian PTEN, suggesting a comparatively close genetic relationship with PTEN (termed MoPTEN). However, the *MoPTEN* gene could functionally complement the *ScTEP1* deletion mutant from resistant phenotype to sensitive phenotype against 20 mM wortmannin added in solid media (Figure 1F). These results indicated that *MoPTEN* is a homolog of *TEP1/PTEN*, and S28515 is the PTEN gene disrupted mutant.

### Expression, Alternative Splicing, and Dual-Phosphatase Activity in MoPTEN

**FIGURE 2:**
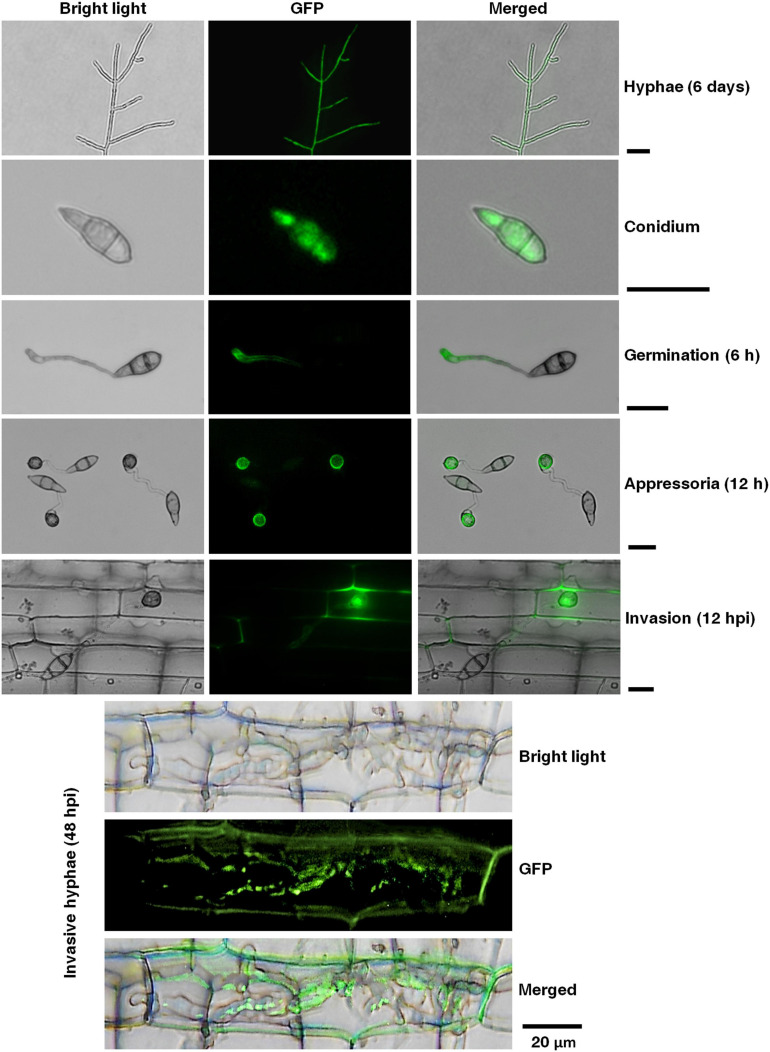
Temporal and spatial dynamics of the *MoPTEN* gene expression in *M. oryzae*. Green fluorescence signals of the MoPTEN-GFP protein were examined by epifluorescence (GFP) microscopy in different developmental stages of *M. oryzae*. All the hyphae, conidia, appressoria and invasive hyphae in leaf-sheath cells were transferred to cover slips before green fluorescence signals observation. The 6-day-old hyphae grown on potato dextrose agar (PDA), conidia harvested from the 10-day-old colonies grown on oatmeal-tomato agar (OTA) media, germinated conidia (6 h), appressoria (12 h), invasive hyphae at 12 h post-inoculation (hpi), and invasive hyphae at 48 hpi were used for green fluorescence signals observation on Nikon Eclipse Ni-U microscope (Nikon, Tokyo, Japan). Scale bar = 20 μm.

To determine the expression pattern, regulation, and localization of the *MoPTEN* gene, a *MoPTEN-GFP* fusion gene driven by its native promoter was transferred into the wild type (Supplementary Figure 1). Fluorescent microscopic observation was carried out in the growing hyphae (6 days), conidia, germinated conidia with germ tube (6 h), appressoria (12 h), and invasive hyphae (12, 48 hpi). Green fluorescence signals of the MoPTEN-GFP protein were detected with slightly weak in growing hyphae and rather strong in conidia, appressoria and invasive hyphae (Figure 2). By comparison, in the wild type or untransformed strains, the background green fluorescence was too weak to be detected (data not shown). This result suggests that MoPTEN expression is upregulated with the development of the infection process of *M. oryzae*.

Semi-qRT-PCR was performed using total RNA isolated from the different growing mycelia (4, 6, 8 days), newly produced mature conidia, germinated conidia, developing appressoria (6, 12, 24 h), and infected leaves with growing invasive hyphae (24, 48, 72 hpi). The results showed the *MoPTEN* gene expression gradually increased and reached a peak in the 12-h developed appressoria, and then decreased and disappeared until at infection stage (Supplementary Figure 5A). Unexpectedly, according to the ORF length of *MoPTEN* (1,977 bp), there appeared an additional band below the *MoPTEN* gene in the developing appressoria and invasive hyphae. In addition, the lower band gradually became the main band as the upper band disappeared within 72 hpi (Supplementary Figure 5A).

**FIGURE 3:**
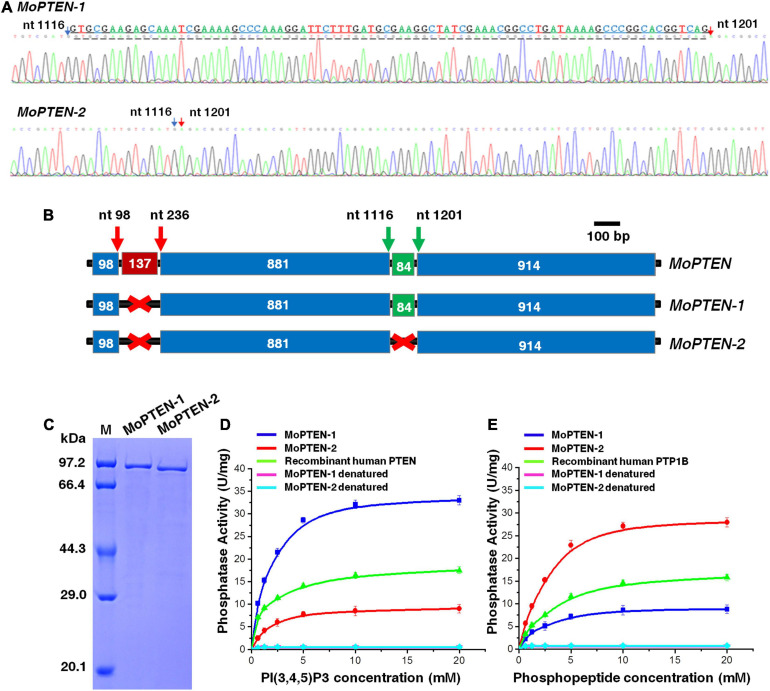
Identification of two alternative splicing variants of *MoPTEN*. **(A)** Sequencing of two transcripts of the *MoPTEN* gene. The partial sequencing results of the two transcripts were shown. The retained intron is underlined, and the numbered arrows indicate two ends of an exon. **(B)** Schematic representation of the *MoPTEN* gene and its splice variants. Alternative splicing occurs in the second intron of *MoPTEN*. The retention or splicing of the second intron leads to two transcript forms, *MoPTEN-1* and *MoPTEN-2*. The upper number stands for the two ends of an exon. **(C)** MoPTEN-1/2 expressed in *Escherichia coli*. Purified both MoPTEN-1/2 were indicated in SDS-PAGE. **(D**,**E)** Measurement of lipid and protein phosphatase activity. PIP3 and phosphopeptide were used as substrates for activity measurement, respectively; the denatured recombinant proteins were used as negative control, and the recombinant human PTEN and PTP1B were used as positive controls. Error bars represent ± SD of three independent repeated samples.

In order to clarify the additional band, the two bands were excised and sequenced, respectively. The results indicated that both sequences were all derived from the *MoPTEN* gene (Figure 3A). The upper band (*MoPTEN-1*) is 1,977 nt long, which is the previously deduced ORF of *MoPTEN* gene; and the lower band (*MoPTEN-2*) is a truncated fragment of *MoPTEN*. In comparison with the DNA sequence of *MoPTEN*, *MoPTEN-2* was 84 nt shorter than *MoPTEN-1*, in which a fragment (between 1,116 and 1,201 nt) was spliced (Figure 3B). The two ends of the 84 nt fragment also have the classic splicing sites “GU-AG,” suggesting *MoPTEN-1* is an intron retention transcript form.

Compared with the *MoPTEN-1*, the *MoPTEN-2* gene encodes a protein of 630 amino acid residues with a predicted molecular mass of 69.4 kDa. The three-dimensional (3D) structures of MoPTEN-1 and MoPTEN-2 were predicted using the web-based server I-TASSER^3^. Both proteins form an unusually deep and wide pocket (Supplementary Figure 5B), which allows PTEN to accommodate the bulky phosphatidylinositol 3,4,5-trisphosphate substrate (Lee et al., 1999). In the pocket, two domains of PTEN, a protein tyrosine phosphatase (PTP) domain and a C2 domain, constitute a single unit; and the PTP domain contains the conserved motif “HCKAGKGR.” The main difference between two forms is that MoPTEN-1 has a calcium ion binding site (A_184_-F_186_-K_220_-E_305_), but MoPTEN-2 has a zinc ion binding site (V_53_- D_57_) (Supplementary Figure 5B). The different ion binding sites may affect the activity and stability of both proteins, and eventually, lead to difference in biochemical properties.

As a dual-specificity phosphatase, PTEN catalyzes the dephosphorylation of protein and lipid substrates and preferentially dephosphorylates PIP3 to PIP2 (Ramaswamy et al., 1999). Recombinant proteins of MoPTEN-1 and MoPTEN-2 were expressed in *E. coli* and purified to homogeneity with a single-step process using a Ni^2+^-NTA column (Figure 3C). Lipid or PTP-specificity phosphatase activity of MoPTEN-1 and MoPTEN-2 was detected with PIP3 or phosphorylated polypeptide Ac-DADE(pY)LIPQQG-NH_2_ as substrate (GENMED Scientifics, Shanghai, China). As a result, MoPTEN-1 activity was significantly higher than that of MoPTEN-2 and the positive control (recombinant human PTEN) when PIP3 was used as substrate (Figure 3D). On the contrary, when the substrate was replaced by phosphorylated polypeptide, MoPTEN-2 activity was significantly higher than that of MoPTEN-1 and the positive control (recombinant human PTP-1B) (Figure 3E). Collectively, these results suggest that alternatively spliced MoPTEN variants possess the distinct expression patterns and dual-phosphatase activity.

### *MoPTEN-1* Is Important for Conidium and Appressorium Formation

To investigate the roles of *MoPTEN-1* and *MoPTEN-2*, we genetically created the knockout mutant strains of *MoPTEN* (Δ*MoPTEN*) (Supplementary Figure 2). Base on Δ*MoPTEN*, we further created three complemented strains harboring Δ*MoPTEN* and the *MoPTEN* gene (Δ*MoPTEN*/*MoPTEN*), the *MoPTEN-1* gene with two synonymous mutations at T^981^A and A^1062^T (Δ*MoPTEN*/*MoPTEN-1*, which cannot be further spliced), and the *MoPTEN-2* gene (Δ*MoPTEN*/*MoPTEN-2*) (Supplementary Figure 3).

**FIGURE 4:**
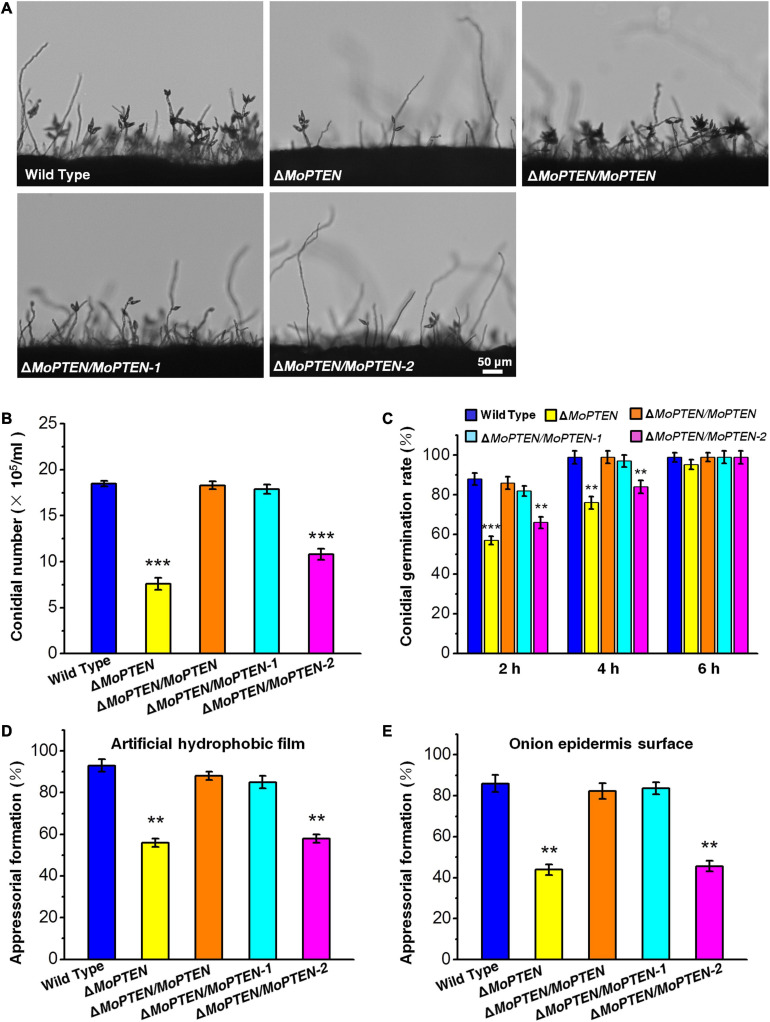
Conidium and appressorium development analysis of the wild type and created strains. **(A)** Conidia formation on conidiophores. Conidia of the wild type and the four created strains (Δ*MoPTEN*, Δ*MoPTEN/MoPTEN*, Δ*MoPTEN/MoPTEN-1*, and Δ*MoPTEN/MoPTEN-2*) from 10-day-old OTA were transferred to cover slips, induced for 48 h, and observed and counted under a light microscope at room temperature. **(B)** Statistical analysis of conidial productivity. The conidia were harvested from the 10-day-old colonies grown on OTA media, and counted using a hemocytometer for all the 5 strains. **(C)** Conidial germination rate. Conidial germination was measured on a hydrophobic surface (plastic cover slips or gel-bond films) and onion epidermal cells and was calculated under the microscope at 2, 4, and 6 hpi. **(D**,**E)** Appressorial formation rate. Appressorial formation was measured on a hydrophobic cover slips and onion epidermis surface and was calculated under the microscope at 12 hpi. Error bars represent ± SD of three independent repeated samples. Two asterisks (**) represent an extremely significant difference at 0.001 < *P* < 0.01, and three asterisks (***) represent an extremely significant differences at *P* < 0.001. Scale bar = 50 μm.

The growth and development of these strains were assessed. When *M. oryzae* was cultivated on PDA and CM plates at 25°C, all the four created strains grew at a rate similar to that of the wild type, and their colony morphologies exhibited little difference (Supplementary Figures 6A,B). However, the deletion of *MoPTEN* significantly decreased conidial production (Figures 4A,B). The complementation of Δ*MoPTEN* with either *MoPTEN* or synonymously mutated *MoPTEN-1* could reverse conidia production to the wild-type level, but Δ*MoPTEN*/*MoPTEN-2* could not (Figures 4A,B). The conidium germination rate of all strains including the wild type was similar at 6 h, although Δ*MoPTEN* and Δ*MoPTEN*/*MoPTEN-2* appeared to be slow in conidium germination at 2 h (57 ± 2.1%, 66 ± 3%) and 4 h (76 ± 3.1%, 84 ± 3.2%) (Figure 4C). In terms of appressorium formation, Δ*MoPTEN*/*MoPTEN* and Δ*MoPTEN*/*MoPTEN-*1 had the formation rate similar as the wild type did; but Δ*MoPTEN* and Δ*MoPTEN*/*MoPTEN-2* were severely affected when induced by artificial hydrophobic film (Figure 4D) or by onion epidermis surface (Figure 4E). These data suggest that *MoPTEN*, in the form of *MoPTEN-1* (the second intron cannot be spliced), is especially involved in fungal growth and development prior to plant infection.

### *MoPTEN-2* Is Important for Invasive Hyphal Growth in Rice Cells

**FIGURE 5:**
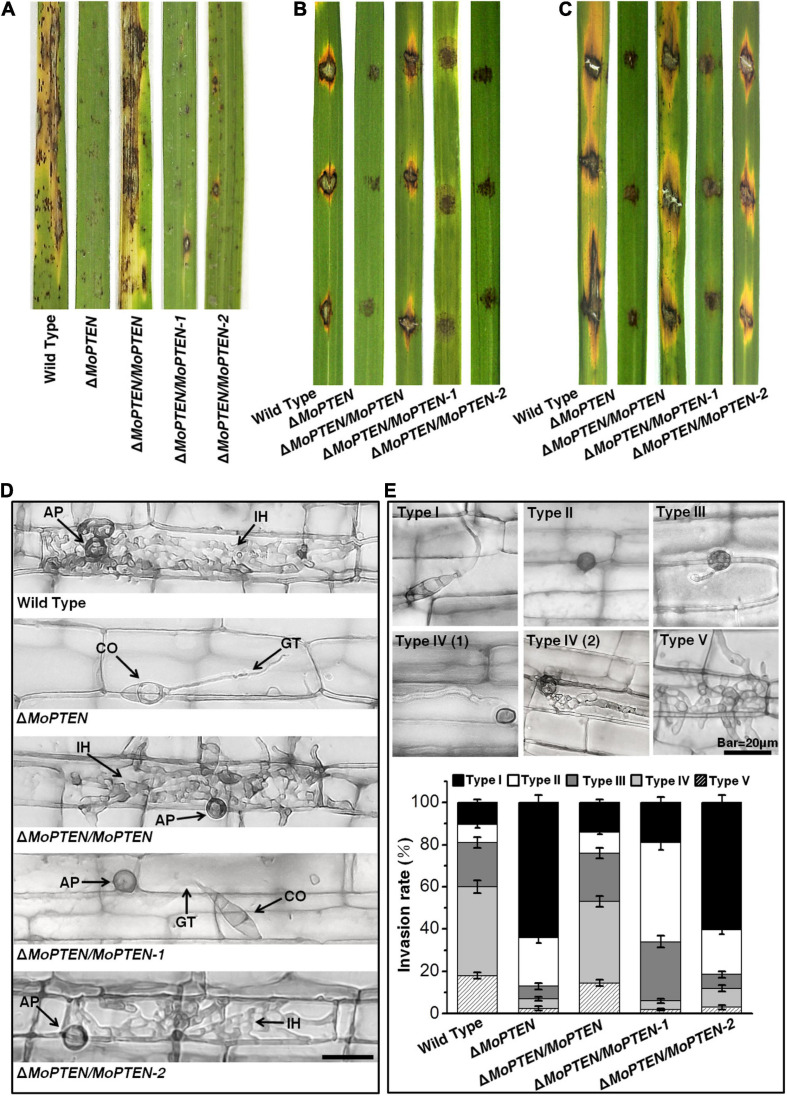
Pathogenesis analysis of the wild type and created strains. **(A)** Spray-inoculation assay. Disease symptoms at 7 dpi of leaves by spraying with conidia (1 × 10^5^/mL). **(B)** Drop-inoculation on unwounded leaves. Disease symptoms following the inoculation of rice leaves with 10-μL droplets of conidia (1 × 10^5^/mL). Representative leaves were photographed at 7 dpi. **(C)** Drop-inoculation on abraded leaves. Disease symptoms following the inoculation of rice leaves with 10-μL droplets of conidia (1 × 10^5^/mL). Representative leaves were photographed at 7 dpi. **(D)** Rice leaf sheath infection assay. The conidial suspension of indicated strains was dropped onto a rice sheath. Representative photographs of invasive hyphae were taken after 48 h of incubation at 25°C. Scale bar = 20 μm. IH, invasive hyphae; CO, conidium; GT, germ tube; AP, appressorium. **(E)** The infection rate was calculated according to the number of type I to type V events. The infection status of more than 100 germinated conidia per leaf sheath was scored at 48 hpi. The experiments were repeated three times and error bars represent ± SD of three independent repeated samples.

In order to characterize the function of *MoPTEN* in pathogenic development, pathogenicity assays were carried out using conidia collected from the four created strains and the wild type. When intact susceptible rice seedlings were spray-inoculated, at 7 dpi, some acute expansive disease lesions were formed in rice leaves by the wild type and Δ*MoPTEN*/*MoPTEN*; but no expansive but restricted lesions were formed in rice leaves by the Δ*MoPTEN*, Δ*MoPTEN*/*MoPTEN-1* and Δ*MoPTEN*/*MoPTEN-2* strains (Figure 5A; Supplementary Figure 6C). Similarly, when drop-inoculation was assayed, only the wild type and Δ*MoPTEN/MoPTEN* strains still showed pathogenicity (Figure 5B). Interestingly, when abraded leaves were drop-inoculated, Δ*MoPTEN*/*MoPTEN-2* caused the similar size lesions as the wild type or Δ*MoPTEN*/*MoPTEN* did (Figure 5C), suggesting the Δ*MoPTEN*/*MoPTEN-2* is incompetent in rice penetration, but competent in invasive hyphal growth in rice cells.

Leaf sheath infection assays were performed to examine the infection effects of the *MoPTEN* and its splice variants in rice host. At 48 hpi, the majority of appressoria of the wild-type and Δ*MoPTEN*/*MoPTEN* invaded rice cells and formed invasive hyphae, but most of the mutant Δ*MoPTEN* did not due to its defect in appressorium formation (Figure 5D). Although Δ*MoPTEN*/*MoPTEN-1* was like the wild type in appressorium formation and the well-developed appressoria appeared to be able to penetrate rice cells, the primary invasive hyphal growth was restricted around infection site (Figure 5D). Although partial appressoria of Δ*MoPTEN*/*MoPTEN-2* were restricted in formation, the normally developed appressoria could then develop into invasive hyphae (Figure 5D).

To decipher the exact action of *MoPTEN* during pathogenic development, we defined the five types of invasive hyphae according to their developmental morphologies (type I, conidia with germ tube; type II, mature appressoria; type III, primary hyphae formed; type IV(1/2), invasive hyphae extended and branched in one cell; type V, invasive hyphae crossing to neighboring cells). Then we quantified the proportion of the five types of invasive hyphae based on 100 germinated conidia in the inoculated leaf sheath (Figure 5E). As a result, more than 80% of inoculated conidia from wild type (89.5 ± 4.4%), Δ*MoPTEN*/*MoPTEN* (86.0 ± 4.0%), and Δ*MoPTEN*/*MoPTEN-1* (81 ± 4.6%) formed mature appressoria, further indicating *MoPTEN-1* is responsible for appressorium development. Most appressoria of the wild type (60.0 ± 3.4%) and complementation strain Δ*MoPTEN/MoPTEN* (53.0 ± 2.9%) could form invasive hyphae of type IV and V, but this situation is only 6.0 ± 1.1% for Δ*MoPTEN*/*MoPTEN-1*. Of the germinated conidia in Δ*MoPTEN*/*MoPTEN-1*, 47.0 ± 3.5% did not form invasive hyphae after forming mature appressoria, and 28.0 ± 2.5% formed growth-restricted primary hyphae (type III), suggesting *MoPTEN-1* has little effect on the development of invasive hyphae. Similar to the mutant (64.0 ± 3.5%), most of the germinated conidia in Δ*MoPTEN*/*MoPTEN-2* (60.5 ± 3.1%) cannot form mature appressoria, however, the proportion of Δ*MoPTEN*/*MoPTEN-2* can form appressoria and continue to develop into invasive hyphae type IV and V has reached 12.0 ± 1.8%, which is about twice that of the mutant Δ*MoPTEN* (7.0 ± 1.4%) and Δ*MoPTEN*/*MoPTEN-1* (6.0 ± 1.1%) (Figure 5E), and these results also indicate the importance of *MoPTEN-2* for invasive hyphae growth in plant cells.

### *MoPTEN-2* Is Crucial for Scavenging Exogenous and Plant Endogenous H_2_O_2_

**FIGURE 6:**
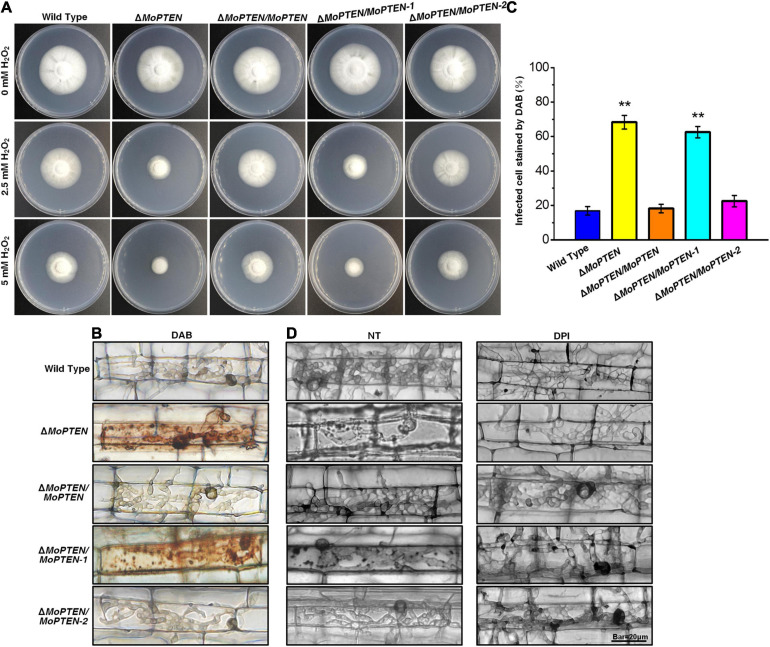
H_2_O_2_ stress assay, DAB staining and DPI treatment of the wild type and created strains. **(A)** Mycelium growth assay. The five strains indicated were cultured on CM supplemented with or without 2.5 mM or 5 mM H_2_O_2_ for 7 days. **(B)** The 3,3′-diaminobenzidine (DAB) staining of leaf sheath cells of rice infected by wild type, mutant and complementation strains at 48 hpi. **(C)** Statistical analysis of DAB staining of leaf sheath cells infected by different strains. **(D)** Rice leaf sheaths were inoculated with conidial suspension (1 × 10^5^ conidia/mL) of wild type, mutant and complementation strains after treatment without (NT) or with 0.4 μM diphenyleneiodonium (DPI) at 48 hpi. The experiments were repeated three times and error bars represent the ± SD of three independent repeated samples for each strain. Two asterisks (**) represent an extremely significant difference at 0.001 < *P* < 0.01. Scale bar = 20 μm.

Mutant S28515 was sensitive to 2.5 mM exogenous H_2_O_2_ (Figure 1A), thus we predict *MoPTEN* is associated with resistance to H_2_O_2_. The four created strains and the wild type were cultivated on CM plates supplemented with H_2_O_2_. We found that the addition of 2.5 mM or 5.0 mM H_2_O_2_ to the growth media seriously inhibited the growth of the Δ*MoPTEN* and Δ*MoPTEN*/*MoPTEN-1* strains in comparison with the remained strains (Figure 6A), suggesting that *MoPTEN-2, not MoPTEN-1*, is able to rescue the defect of Δ*MoPTEN* in H_2_O_2_ resistance.

As rice plant accumulates more H_2_O_2_ during pathogen-rice interaction, and *MoPTEN-2* expression increases with pathogenic development of *M. oryzae*, we speculate that MoPTEN-2 is responsible for the clearance of host-derived H_2_O_2_ during infection. To test this, DAB staining was used to identify the endogenous ROS accumulated in the cells of rice leaf sheath infected by *M. oryzae* at 48 hpi (Figure 6B). In the leaf sheaths inoculated by the Δ*MoPTEN* and Δ*MoPTEN*/*MoPTEN-1* strains, more than 80 and 50% of the infected cells investigated were stained dark brown, respectively; in contrast, less than 25% of the infected cells were stained light brown or colorless as wild type, Δ*MoPTEN*/*MoPTEN* and Δ*MoPTEN*/*MoPTEN*-2 (Figures 6B,C), displaying loss of H_2_O_2_ scavenging function in Δ*MoPTEN* and Δ*MoPTEN*/*MoPTEN-1.* These results reveal that *MoPTEN-2* is crucial for scavenging exogenous and plant-derived H_2_O_2_.

In order to further verify whether the restricted development of the invasive hyphae of the mutant is related to the inability to scavenge the H_2_O_2_ produced by host cells, we used NADPH oxidase inhibitor DPI to treat conidia before infecting leaf sheaths. After incubation at 25°C for 48 h, the infected rice leaf sheath cells were observed through an optical microscope. Without treatment of DPI, the growth and development of the invasive hyphae of Δ*MoPTEN* and Δ*MoPTEN*/*MoPTEN-1* was restricted, and granular deposits could be observed in the infected cells, while those of wild type, Δ*MoPTEN*/*MoPTEN* and Δ*MoPTEN*/*MoPTEN-2* could grow normally and extend to adjacent cells (Figure 6D). After inhibiting the production of ROS in host cells with DPI, the invasive hyphae of Δ*MoPTEN* and Δ*MoPTEN*/*MoPTEN-1* could also grow normally and extend to neighboring cells. Meanwhile the granular deposits in Δ*MoPTEN* and Δ*MoPTEN*/*MoPTEN-1*-infected rice cells decreased (Figure 6D). This result also proved that the *MoPTEN*, in the form of *MoPTEN-2*, participates in the process of removing host-generated ROS, and this may also be an important reason for the decreased virulence and the restriction of the growth and development of invasive hyphae.

## Discussion

PTEN has been extensively studied in humans and mammals (Malaney et al., 2017). To suppress tumor effectively, PTEN must be expressed in a normal level and pattern (Abou Faycal et al., 2016; Malaney et al., 2017). However, PTEN and its homologs are often regulated by alternative splicing and formed aberrant variants (Agrawal and Eng, 2006; Sarquis et al., 2006; David and Manley, 2010). Different from the human PTEN, fungal homologs play roles in sporulation and pathogenesis (Di Stasio et al., 2009; Zhang et al., 2010; Vijayakrishnapillai et al., 2018), but the relationships between the fungal PTEN and alternative splicing are largely unknown. In this research, we identified a filamentous fungal homolog of PTEN in a model phytopathogen *M. oryzae*. The *MoPTEN* was associated with H_2_O_2_ resistance and pathogenicity in the blast fungus. Alternative splicing occurred in the second intron results in the intron retained form MoPTEN-1 and spliced an isoform MoPTEN-2.

Generally, PTEN and its homologous proteins contain the conserved N-terminal PTP catalytic domain (Haynie and Xue, 2015). Amino acid sequence analysis reveals that *MoPTEN* is closely associated with the filamentous fungal homologs of *PTEN*, such as *FgTEP1* (Figure 1E; Supplementary Figure 4B). As a lipid and protein phosphatase, PTEN is a non-redundant negative regulator of the PI3K/AKT pathway. Therefore, yeast cells deleted for *TEP1/PTEN* are resistant to the PI3K inhibitor wortmannin (Heymont et al., 2000). The *MoPTEN* could functionally rescue the defective phenotype of the *TEP1*/*PTEN* deletion strains of yeast, confirming that *MoPTEN* has the basic biological functions of *PTEN* that depends on the lipid phosphatase activity. On the other hand, MoPTEN lacks of several conserved domains, such as PDZ and PEST in the Carboxyl-terminal of PTEN (Supplementary Figure 4A) although MoPTEN has the similar pocket-shaped structure to human PTEN (Supplementary Figure 5B). In addition, the retention or exclusion of the second intron sequence (84 nt) caused the changes of metal ion binding sites (Supplementary Figure 5B), suggesting that MoPTEN is a distinctive homolog of PTEN.

**FIGURE 7:**
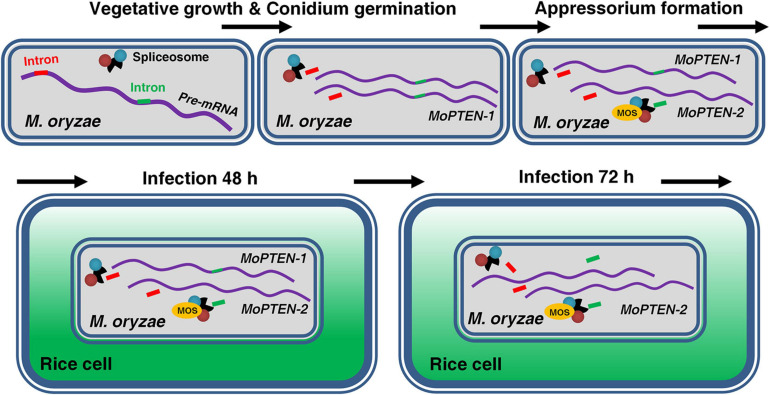
Alternative splicing of *MoPTEN*. *MoPTEN* alternative splicing variants were formed in a phased manner, which, respectively, played a phased role. *MoPTEN-1*, with the second intron retained, was expressed mainly in conidia and appressoria, and the spliced form *MoPTEN-2* was expressed mainly in growing invasive hyphae.

More alternative splicing cases occurred in PTEN and homologs have been reported in humans (Agrawal and Eng, 2006; Sarquis et al., 2006; David and Manley, 2010; Malaney et al., 2017), suggesting the human PTEN is more susceptible to alternative splicing; but *PTEN* alternative splicing has never been described in fungi before this research. For the first time we discovered a case of alternative splicing occurred in *PTEN* gene in *M. oryzae* (Figures 3A,B; Supplementary Figure 5A). In addition, both splice variants were expressed in a relay manner: the previously deduced *MoPTEN-1*, with the second intron retained, was expressed mainly in conidia and appressoria (fungal development pre-plant infection); and the spliced form *MoPTEN-2* was expressed mainly in growing invasive hyphae (fungal development post-plant infection) (Figure 7).

Although PTEN contains the dual specificity lipid and protein phosphatase catalytic domain, the most extensively studied tumor-suppressive function of PTEN is its lipid phosphatase activity (Ramaswamy et al., 1999). Both MoPTEN-1 and MoPTEN-2 have the same lipid and protein phosphatase catalytic domain (Figures 1D,E); however, they are different in the catalytic substrates: MoPTEN-1 preferred to catalyze lipid substrates but MoPTEN-2 preferred to catalyze phosphorylated proteins (Figures 3D,E). The addition of exogenous H_2_O_2_ or the intracellular production of this metabolite in response to certain stressors affects the activities of phosphatases (Finkel, 1998). Exposure of purified PTEN or of cells to H_2_O_2_ resulted in inactivation of PTEN (Lee et al., 2002; Kwon et al., 2004). Therefore, we analyzed the activities of both MoPTEN-1/2 proteins in response to H_2_O_2_ treatment. The activity of MoPTEN-2 was not affected significantly, but MoPTEN-1 was inhibited by up to 80% when treated using 0.4 mM H_2_O_2_ (Supplementary Figure 7), indicating that MoPTEN-2 is more stable than MoPTEN-1 and crucial under H_2_O_2_ stress derived in pathogen-host interaction.

As a second messenger, PIP3 is involved in multiple physiological processes in many eukaryotic cells (Falasca and Maffucci, 2009). In *Phytophthora* pathogens, PIP3 is required for full virulence (Lu et al., 2013), and mediates the entry of eukaryotic pathogen effectors into plant and animal host cells (Kale et al., 2010). PTEN dephosphorylates the secondary messenger PIP3 to PIP2, and then regulates cell proliferation, differentiation, and survival by blocking the PI3K/AKT pathway (Gil et al., 1999; Huang et al., 1999; Solari et al., 2005; Serezani et al., 2012; Liu and Chin-Sang, 2015). Therefore, it seems that the dephosphorylation activity of PTEN should be detrimental to oomycete pathogenesis due to the shift of PIP3 to PIP2. However, in this study, MoPTEN-1, with high lipid phosphatase activity, were expressed in high levels during conidium and appressorium development, implying MoPTEN-1 promotes plant infection. Considering the differences between *Ascomycetes* and *Oomycetes*, it raises a possibility that *M. oryzae* probably adopts a different pathogenic pathway from *Phytophthora* species, in which MoPTEN-1 is responsible for the balance of PIP3 and PIP2, and then suitable for plant infection. Introns are mediators of cell response to starvation (Parenteau et al., 2019), MoPTEN-1 may be involved in second metabolic regulation, especially the synthesis of melanin (Supplementary Figure 8A) due to the intron retention, for we know melanin is one of the most important factors for appressoria differentiation and infection (Kawamura et al., 1997; Manfiolli et al., 2019; Harata et al., 2020). In this research we found that MoPTEN-1 could rescue the defect of Δ*MoPTEN* in melanin production (Supplementary Figure 8A); and actually, several melanin synthesis related genes were upregulated in Δ*MoPTEN/MoPTEN-1* (Supplementary Figure 8B).

In plant fungal pathogens, reversible protein phosphorylation by protein kinase, such as the central factors of MAP kinase (MAPK), calcium, and cAMP signaling pathways, is a major mechanism for the regulation of pathogenic development (Lee et al., 2003; Zhao et al., 2007; Nguyen et al., 2008; Manfiolli et al., 2019). Quantitative evidence indicates that PTPs are also key regulators in pathogenic signaling transduction (Lee and Levin, 2018; Liu and Levin, 2018). The dual specificity MAPK phosphatase, Rok1, is involved in mating, filamentation, appressorium formation and subsequent disease development by regulating the activities of Kpp2 and Kpp6 in *Ustilago maydis* (Di Stasio et al., 2009). In another case, loss of *FgTEP1* reduces pathogenicity in *Fusarium graminearum* (Zhang et al., 2010). Recently, the requirement of *TEP1/PTEN* homolog has also been shown in virulence of *U. maydis* and *Colletotrichum graminicola* (Vijayakrishnapillai et al., 2018; Wang et al., 2021). Consistently, deletion of the *MoPTEN* gene caused reduced pathogenicity in *M. oryzae* (Figures 5A,B). Individual splice variants of *MoPTEN* could not rescue the defect of virulence in Δ*MoPTEN* (Figures 5A,B) although MoPTEN-2 contains protein phosphatase activity (Figure 3E). However, when the wounded leaves were used for inoculation, Δ*MoPTEN/MoPTEN-2* virulence was restored, reflecting its defect in plant penetration (Figure 5C). Once the pathogen entering plant cells, partial intracellular hyphae enable grow and extend in host cells (Figures 5C,D,E).

Protein phosphatase activity of MoPTEN-2 is stable to H_2_O_2_ treatment (Supplementary Figure 7). Δ*MoPTEN/MoPTEN-2*, like the wild type or complementation strain Δ*MoPTEN/MoPTEN*, was resistant to H_2_O_2_ (Figure 6A). Therefore, we propose that the stable *MoPTEN-2* is also capable of scavenging host-derived ROS. Indeed, in Δ*MoPTEN/MoPTEN-2* infected rice cells, DAB staining signals were hard to be detected, but strong in Δ*MoPTEN* and Δ*MoPTEN/MoPTEN-1* (Figures 6B,C). In Δ*MoPTEN* and Δ*MoPTEN/MoPTEN-1* strains, several selected degradation genes of H_2_O_2_ were significantly downregulated, but Δ*MoPTEN/MoPTEN-2* maintained a level as high as the wild type or Δ*MoPTEN/MoPTEN* (Supplementary Figure 9). In addition, after being treated by DPI, the invasive hyphae of Δ*MoPTEN* and Δ*MoPTEN*/*MoPTEN-1*, which are restricted in growth and development without treatment of DPI, can grow normally (Figure 6D). The above results suggest that *MoPTEN-2* is realted in regulation of ROS degradation.

The pathogenic process of the rice blast fungus can be divided into two phases: pre-plant and post-plant infection, while the later also includes biotrophic growth and necrotrophic growth. Conidium and appressorium formation occurs outside plant cells, and invasive hyphae extension occurs inside host (Talbot, 2003; Kankanala et al., 2007). For a successful infection cycle, *MoPTEN-1* and *MoPTEN-2* served pathogenic development of *M. oryzae* in a relay model (Figure 7). According to our research results, it can be seen that *MoPTEN-1* mainly regulates the conidial formation and development of appressorium of *M. oryzae*, while *MoPTEN-2* plays a dominant role in the development and extension of invasive hyphae. Therefore, we analyzed that the occurrence of alternative splicing of *MoPTEN* may be affected by some factors in the post-plant infection process. For example, after infecting host cells, Δ*MoPTEN*/*MoPTEN-1*, similar to mutant Δ*MoPTEN*, could not effectively scavenge H_2_O_2_ produced by host cells, while Δ*MoPTEN*/*MoPTEN-2* could eliminate H_2_O_2_ as well as wild type and Δ*MoPTEN*/*MoPTEN* strains (Figures 6B,C). Therefore, ROS produced by plant defense response during pathogen infection may be one of the inducements for alternative splicing of *MoPTEN*. In addition, the alternative splicing process of *MoPTEN* may also be regulated by some other genes in *M. oryzae*. Many studies reported that the survival motor neuron (SMN) protein undergoes alternative splicing, and through which it is involved in cellular activities (Liang et al., 2015). On the other hand, SMN functions in the cytoplasmic assembly of Sm-class snRNPs, core particles of the spliceosome (Pellizzoni et al., 2002; Li et al., 2014; Matera and Wang, 2014), exhibiting a role in regulating pre-mRNA alternative splicing. Reduced levels of SMN protein result in a common neuromuscular disorder (SMA) in humans (Lefebvre et al., 1995; Schrank et al., 1997). In fungi, a SMN homolog is associated with pathogenesis in the disease development of rice blast; particularly, the *SMN/MOS* deletion strains of *M. oryzae* shows similar defects to Δ*MoPTEN* in many aspects, such as fewer sporulation, sensitivity to H_2_O_2_ and reduced pathogenicity (Liang et al., 2015). And because PTEN can interact with the spliceosomal proteins and drive pre-mRNA splicing (Song et al., 2011; Shen et al., 2018), we assume there exists a regulative relationship between MoPTEN and SMN/MOS (Figure 7). Indeed, in the previous research, we found that *MoPTEN* is regulated by *SMN/MOS* through transcriptome sequencing, and we have obtained some research results that the expression of *MoPTEN* in Δ*MOS* is down-regulated from that in Δ*MOS/MOS* (data not shown). The splicing process is carried out by the large spliceosome complex consisting of RNA and proteins (Wahl et al., 2009; Shen et al., 2018). The precise regulation of *MoPTEN* alternative splicing should require more factors than *SMN*, by exploring which we will decipher the regulatory mechanism.

## Data Availability Statement

The original contributions presented in the study are included in the article/Supplementary Material, further inquiries can be directed to the corresponding author/s.

## Author Contributions

S-HZ and YW designed the research. SW performed the research. HL, YD, and PZ assisted in part of the experimental process. SW, GL, and S-HZ analyzed the data. S-HZ and SW wrote the manuscript. All authors contributed to the article and approved the submitted version.

## Conflict of Interest

The authors declare that the research was conducted in the absence of any commercial or financial relationships that could be construed as a potential conflict of interest.

## Footnotes

1 https://blast.ncbi.nlm.nih.gov/Blast.cgi

2 https://www.ncbi.nlm.nih.gov/; http://fungi.ensembl.org/Magnaporthe_oryzae/

3 http://zhanglab.ccmb.med.umich.edu/I-TASSER/

## Abbreviations

CM: complete minimal media
DAB: 3,3′-diaminobenzidine
dpi: days post-inoculation
DPI: diphenyleneiodonium
hpi: hours post-inoculation
OTA: oatmeal-tomato agar
PDA: potato dextrose agar
PIP2: phosphatidylinositol-4,5-diphosphate
PIP3: phosphatidylinositol-3,4,5-trisphosphate
PTP: protein tyrosine phosphatase
ROS: reactive oxygen species.
