# Supplementary material for: Alternative Splicing of MoPTEN Is Important for Growth and Pathogenesis in Magnaporthe oryzae
Source: Front Microbiol. 2021 Jul 16;12:715773. doi: 10.3389/fmicb.2021.715773 (PMC8322540; doi:10.3389/fmicb.2021.715773)
Supplement: Supplementary file 1 [file Data_Sheet_1.DOCX]

Supplementary Material

**Figure S1.** The construction strategies for the *MoPTEN::GFP* strains. **(A)** Structure of the vector pCAMBIA1303. **(B)** Strategy of the construction of the *MoPTEN::GFP* fusion gene vector. The fragment with a DNA length of 3107 bp (the native promoter of *MoPTEN* is included, 1000 bp) amplified from the genomic DNA of wild type JL0910 was ligated into the *Spe*Ⅰ multiple cloning site of the vector pCAMBIA1303, generating a recombinant fusion vector. **(C)** Confirmation of the *HPH* gene. **(D)** Confirmation of the *GFP* gene. **(E)** The expression of *MoPTEN-GFP* fusion gene by qRT-PCR. Details of the construction are described in Materials and methods.

**Figure S2.** The construction strategy for the *MoPTEN* deletion mutants. **(A)** Structure of the knockout vector pXEH 2.0. **(B)** The knockout strategy for the *MoPTEN* gene deletion. M-L: upstream flanking sequence of *MoPTEN*. M-R: downstream flanking sequence of *MoPTEN*. The genomic DNA of the JL0910 strain was amplified for upstream and downstream flanking sequences of *MoPTEN*, which was cloned between the *Eco*RⅠ-*Kpn*Ⅰ and *Xba*Ⅰ-*Sal*Ⅰ sites of the pXEH 2.0 vector generating the replacement vector. *HPH*: hygromycin B phosphotransferase resistance gene. **(C)** Confirmation of the deletion strains by qRT-PCR.

**Figure S3.** The construction strategies for the complementation strains of *MoPTEN*. **(A)** Structure of complementary vector pKD7-Red. PH3: H3 promoter. NEO: neomycin phosphotransferaseⅡ gene. RP27 ter: terminator. **(B)** Strategy for the construction of recombinant vector. The full-length DNA sequence of the *MoPTEN* gene, the *MoPTEN-1* cDNA containing two synonymous mutation sites at T^981^A and A^1062^T, and the *MoPTEN-2* cDNA (second intron spliced form) were amplified and cloned into the two *Sma*Ⅰ sites of the pKD7-Red vector generating the final fusion vectors. **(C)** Confirmation of the target gene of complementation strains by PCR. M, DNA marker; Lane 1, Δ*MoPTEN*/*MoPTEN*; Lane 2, Δ*MoPTEN*/*MoPTEN-1*；Lane 3, Δ*MoPTEN*/*MoPTEN-2*；Lane 4, negative control (water). **(D)** The expression levels of the target genes of complementation strains versus the wild type JL0910 by semi-qRT-PCR.

**Figure S4.** Full-length sequence alignment and phylogenetic tree of the MoPTEN and related homologs**. (A)** Full-length sequence alignment of the MoPTEN and other reported homologs in selected species. The dark blue indicates that the homology of sequences is 100%. The pink indicates homology ≥75%, and the cyan indicates homology ≥50%. The black box represents predicted phosphatase domain; the green box represents the spliced sequence, and the red boxes are marked to show the several C-terminal domains in human PTEN. **(B)** The phylogenetic tree represents the genetic relationship between MoPTEN and its homologous proteins in different species. Use the MUSCLE program and Neighbor-Joining algorithm in the MAGA software^[[1]](#footnote-1)^ to analyze homologous amino acid sequences alignments and construct a phylogenetic tree. MoPTEN, MGG_08005 is marked in red.

**Figure S5.** The expression of two transcripts of *MoPTEN* in different periods of *M. oryzae* and the predicted three-dimensional structure of the protein **(A)** Agarose gel showing the amplification products. Both *MoPTEN-1* and *MoPTEN-2* transcripts were detected by semi-qRT-PCR. mRNA was extracted from different developmental stages of *M. oryzae*, and reversely transcribed into cDNA, then detected by semi-qRT-PCR. **(B)** Three-dimensional structures of MoPTEN-1/2. Both forms showed a typical pocket structure. The catalytic active site and metal ion site are pointed in dotted boxes.

**Figure S6.** Growth and pathogenic analysis of the *M. oryzae* wild type and the four created strains. **(A)** Wild type, mutant and complementation strains were cultured on potato dextrose agar (PDA) or complete minimal (CM) media plates for 9 days at 25°C. The created strains are similar to the wild type in colony phenotype. **(B)** Statistical analysis of the colony diameters of the strains. **(C)** Statistical analysis of disease lesions. Disease lesions were classed six types according to Liu et al. (Liu et al., 2016). The six types of disease lesions were counted and photographed in the leaf area of 4 cm^2^ at 7 days post-inoculation (dpi). The above experiments were performed in triplicate and repeated three independent times for each strain. One asterisk (*) represents an significant difference at 0.01 < *P* < 0.05, and three asterisks (***) represent an extremely significant differences at *P* < 0.001. Error bars represent the ± SD of three independently repeated samples.

**Figure S7.** Stability test of MoPTEN-1 and MoPTEN-2 to H_2_O_2_. Purified recombinant MoPTEN-1 and MoPTEN-2 (150 µM) was incubated for 30 min at 30°C in the presence of different concentrations of H_2_O_2_. The percentage is the ratio of phosphatase activity with and without hydrogen peroxide in the reaction system.

**Figure S8.** Appressorial melanin analysis. **(A)** Melanin was extracted respectively from the conidia and the conidia with formed appressoria. The tested strains were suspended in 10 mL NaOH solution (1 M), respectively. **(B)** The expression levels of genes *ALB1*, *BUF1* and *RSY1* related to melanin formation of *M. oryzae* in the wild type, mutant and complementation strains. The above experiments were performed in triplicate and repeated three independent times for each strain. Error bars represent the ± SD of three independently repeated samples. Two asterisks (**) represent an extremely significant difference at 0.001 < *P* < 0.01.

**Figure S9.** Expression of the H_2_O_2_ degradation genes in the created and wild type strains. MGG_13230, MGG_04404 and Yap1 are involved in H_2_O_2_ degradation. Expression of such genes was quantified using qRT-PCR with cDNA synthesis from the wild-type JL0910 and the four created strains. Error bars represent the ± SD of three independently repeated samples. Two asterisks (**) represent an extremely significant difference at 0.001 < *P* < 0.01.

**Table S1. Primers used for PCR amplification in this research.**

| **Primer Name** | **Purpose** | **Sequence 5′-3′** |
| --- | --- | --- |
| HPH1 | Southern analysis | GTCGGCATCTACTCTATTCC |
| HPH2 | Southern analysis | CCTATTCTACCCAAGCATCC |
| Prom-PTEN-F | *MoPTEN::GFP* expression | GGACTAGTAAGGGTGCACGGATGATG |
| Prom-PTEN-R | *MoPTEN::GFP* expression | GGACTAGTATCAACAATAAACCCCTT |
| Semi-YP-F | qRT-PCR | ATGGCATCACTCCTGCGCCA |
| Semi-YP-R | qRT-PCR | ATCAACAATAAACCCCTTCT |
| Semi-actin-F | qRT-PCR | CTCCCCGCGCCGTCTTCC |
| Semi-actin-R | qRT-PCR | GCTCGTTGTAGAAGGTGT |
| Mq-YP-F | Real time PCR | GCAGAGGCTATTTCTACCAC |
| Mq-YP-R | Real time PCR | GTAGCGGCATTCTTGATCTT |
| Mq-actin-F | Real time PCR | CCATGTACCCTGGTCTTTCG |
| Mq-actin-R | Real time PCR | TTCGAGATCCACATCTGCTG |
| MBD-F | *E. coli* expression | CGGAATTCATGGCATCACTCCTGCGC |
| MBD-R | *E. coli* expression | CCAAGCTTTCAATCAACAATAAACCC |
| MGG-qc-LF | MoPTEN deletion | CGGAATTCTAGGCCACGGAATGAAGGAG |
| MGG-qc-LR | MoPTEN deletion | GGGGTACCCGGCAACCCAAAAAAAGAAG |
| MGG-qc-RF | MoPTEN deletion | GCTCTAGATTTCTCCAGGCTCCTTTTCC |
| MGG-qc-RR | MoPTEN deletion | GCGTCGACTGGCTGCTTTGGTCTGTTCT |
| MoPTEN-F | Mutant identification | TGGGGTGTCGGAGGTGGTAC |
| MoPTEN-R | Mutant identification | GGCTTTGCTTGCTCTGGTTT |
| HYG-YZ-F | Mutant identification | GCCCTTCCTCCCTTTATTTC |
| HYG-YZ-R | Mutant identification | ACTCTATTCCTTTGCCCTCG |
| M-L-F | Mutant identification | AAATCGGGACAACAGCAAC |
| M-L-R | Mutant identification | CAAACGCACCAAGTTATCG |
| MGG-hb-F | Complementation | TCCCCCGGGATGGCATCACTCCTGCGC |
| MGG-hb-R | Complementation | TCCCCCGGGATCAACAATAAACCCCTT |
| GFP-F | PCR identification | AGTAAAGGAGAAGAACTTTT |
| GFP-R | PCR identification | TTTGTATAGTTCATCCATGC |
| ALB1-F | Melanin gene identification | GCAATGTCGGTCCCAACTA |
| ALB1-R | Melanin gene identification | ATCTCAAAGGCGATGACAC |
| BUF1-F | Melanin gene identification | ACGCCGTCTACTCAGGATC |
| BUF1-R | Melanin gene identification | TCTCGCCGTTTGGAATGTA |
| RSY1-F | Melanin gene identification | CGACTCCAAGGACTGGGAT |
| RSY1-R | Melanin gene identification | GTCCTCGGACACCTTCTCC |
| RB1 | Tail-PCR | GGCACTGGCCGTCGTTTTACAAC |
| RB2 | Tail-PCR | AACGTCGTGACTGGGAAAACCCT |
| RB3 | Tail-PCR | CCCTTCCCAACAGTTGCGCA |
| LB1 | Tail-PCR | CCGAGGGCAAAGGAATAGAGTAG |
| LB2 | Tail-PCR | CGACGGTATCGATAATCAGCTGT |
| LB3 | Tail-PCR | CCCCAGTACATTAAAAACGTCCG |
| AD1 | Tail-PCR | TG(A/T)GNAG(A/T)ANCA(G/C)AGA |
| AD2 | Tail-PCR | (A/T)AGTGNAG(A/T)ANCANAGA |
| AD3 | Tail-PCR | (G/C)TTGNTA(G/C)TNCTNTGC |
| AD4 | Tail-PCR | (A/T)CAGNTG(A/T)TNGTNCTG |
| MoPTEN-1(SM)-F | Site-directed mutation | TCGCATCAAAGAATCCTTTGGGCTTTTCGATTTGCTCTTCGCTCCATCG |
| MoPTEN-1(SM)-R | Site-directed mutation | AGGCTATCGAAACGGCCTGATAAAAGCCCGGCACGGTCTGAGACGGCCA |
| Yap1-RT-F | Real time PCR | CCGAGGACTTTACCATCCTC |
| Yap1-RT-R | Real time PCR | CCTTGAGCCACTTGAAGAGC |
| 13230-RT-F | Real time PCR | GTCACAGCGTGACGACAAG |
| 13230-RT-R | Real time PCR | GTCCTCCTGTCGCTTCAGAC |
| 04404-RT-F | Real time PCR | CCACGAGCTCAACTTTGGAT |
| 04404-RT-R | Real time PCR | GGACGGTGACAAGCATCTC |

Note: Sequences which marked with “___” stand for restriction enzyme cutting site

**References**

Liu, X.Y., Qian, B., Gao, C., Huang, S., Cai, Y., Zhang, H., et al. (2016). The Putative Protein Phosphatase MoYvh1 Functions Upstream of MoPdeH to Regulate the Development and Pathogenicity in *Magnaporthe oryzae*. *Mol Plant Microbe Interact* 29(6)**,** 496-507. doi: 10.1094/MPMI-11-15-0259-R.

1. https://www.megasoftware.net/ [↑](#footnote-ref-1)
