# Supplementary figures and images for: Alternative Splicing of MoPTEN Is Important for Growth and Pathogenesis in Magnaporthe oryzae
Source: Front Microbiol. 2021 Jul 16;12:715773. doi: 10.3389/fmicb.2021.715773 (PMC8322540; doi:10.3389/fmicb.2021.715773)

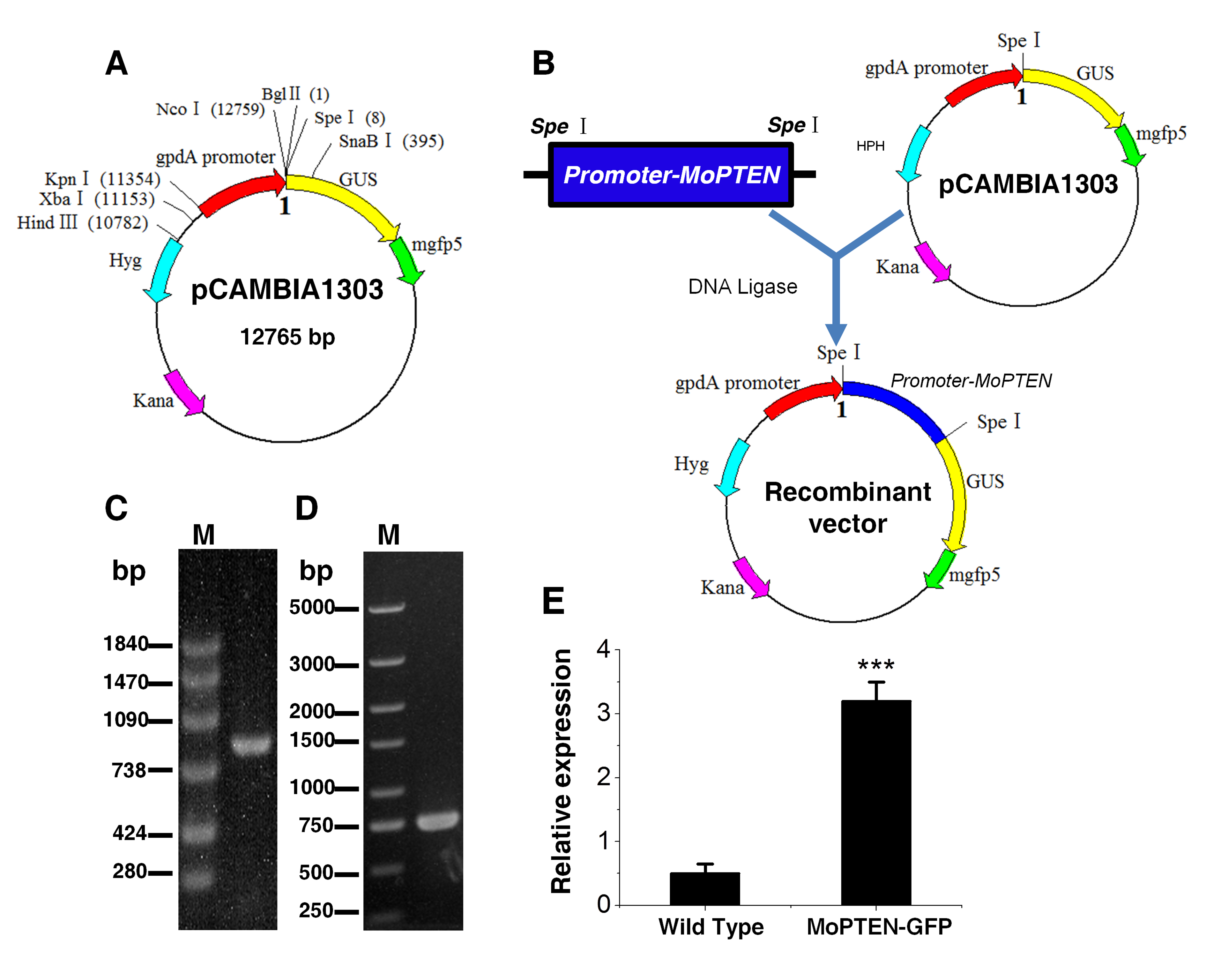

Supplement: Supplementary file 2 [file Image_1.TIF]

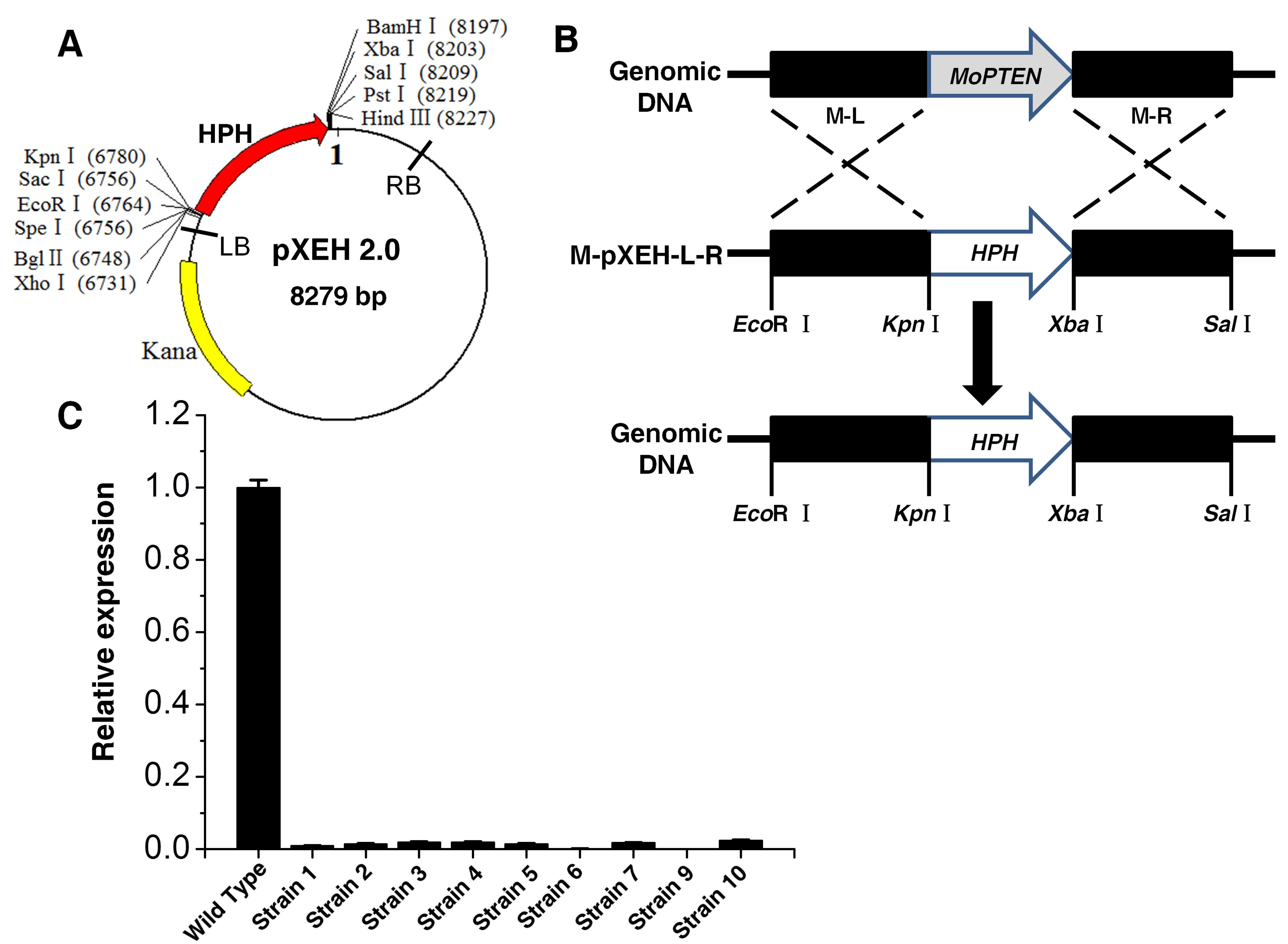

Supplement: Supplementary file 3 [file Image_2.TIF]

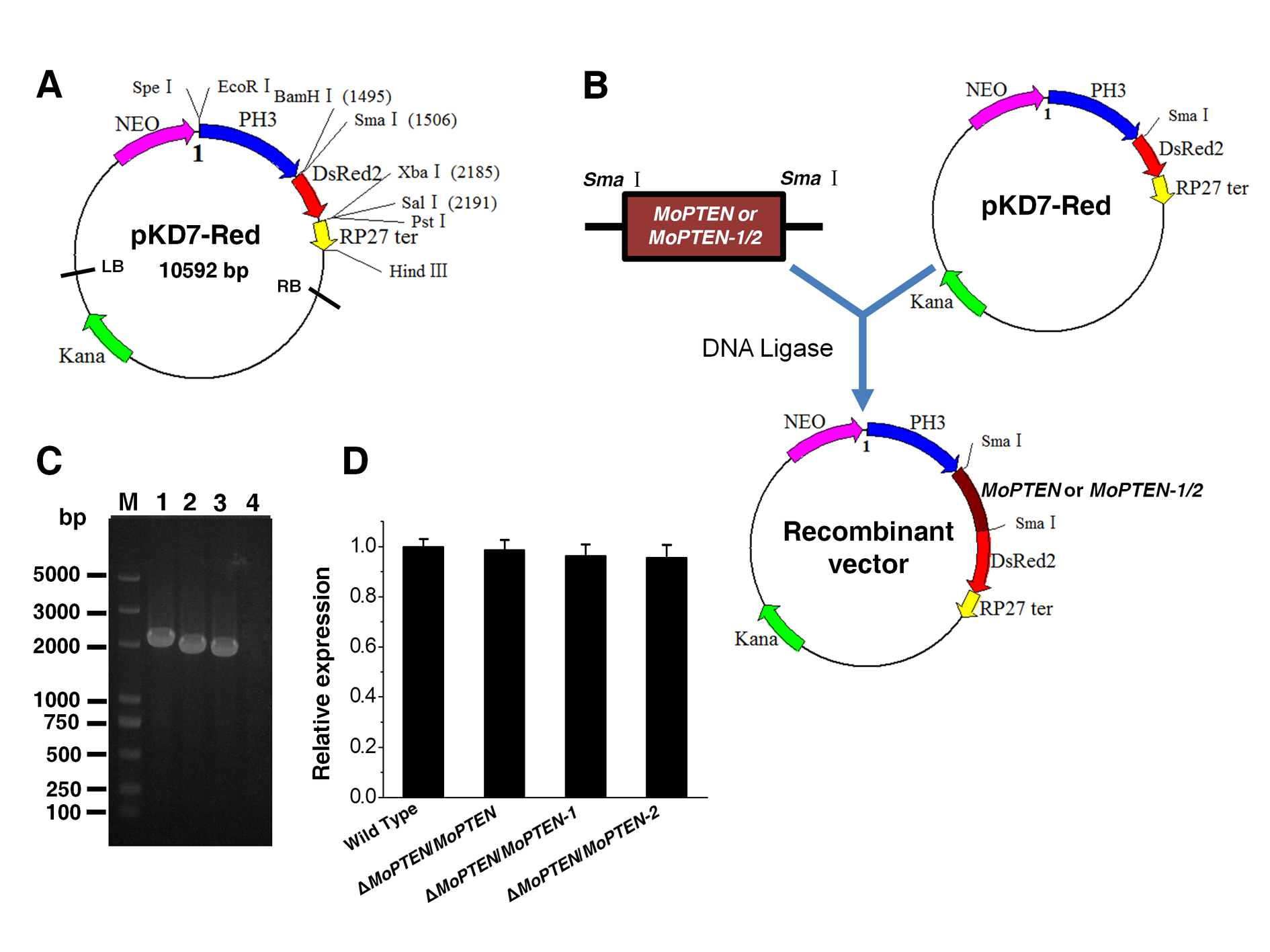

Supplement: Supplementary file 4 [file Image_3.TIF]

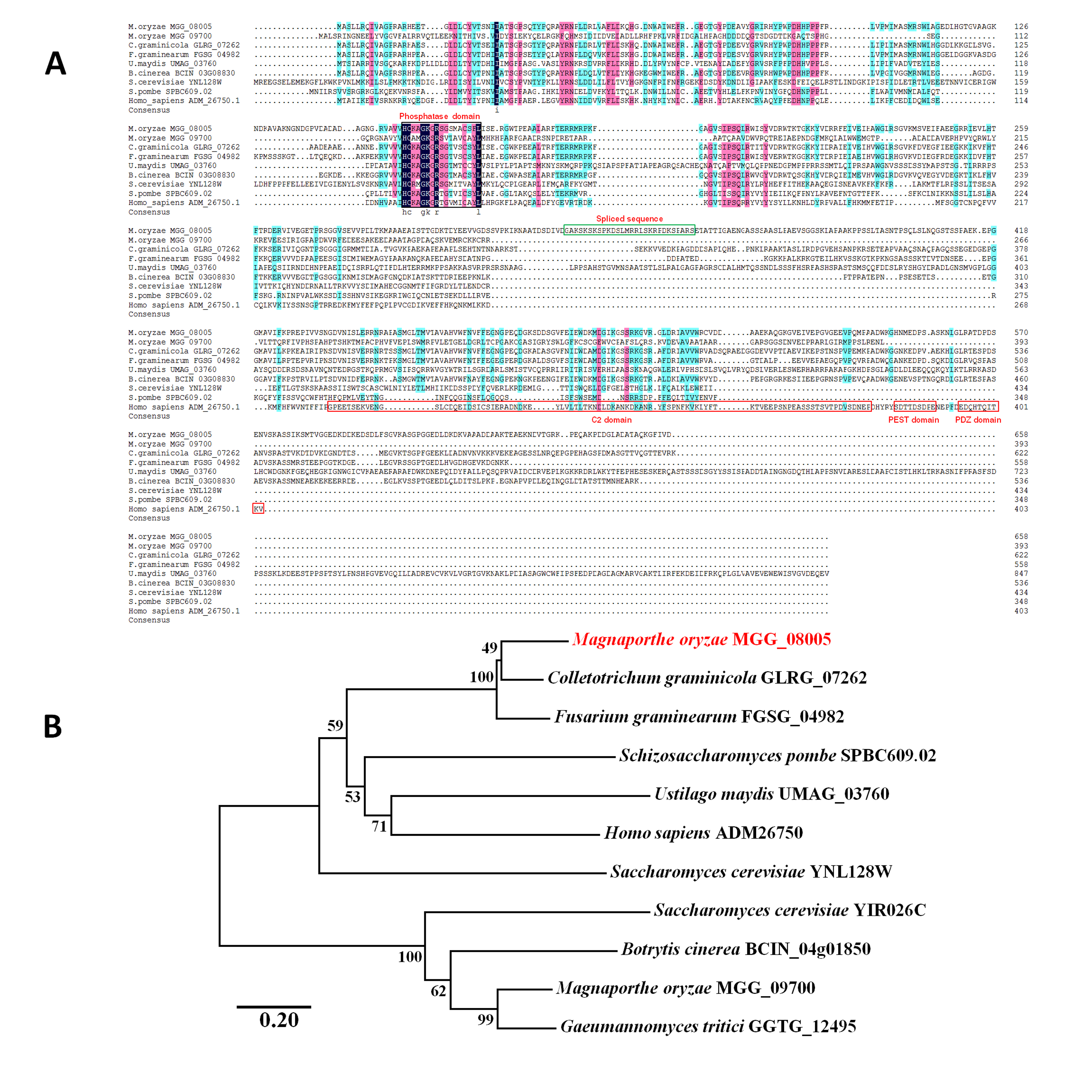

Supplement: Supplementary file 5 [file Image_4.TIF]

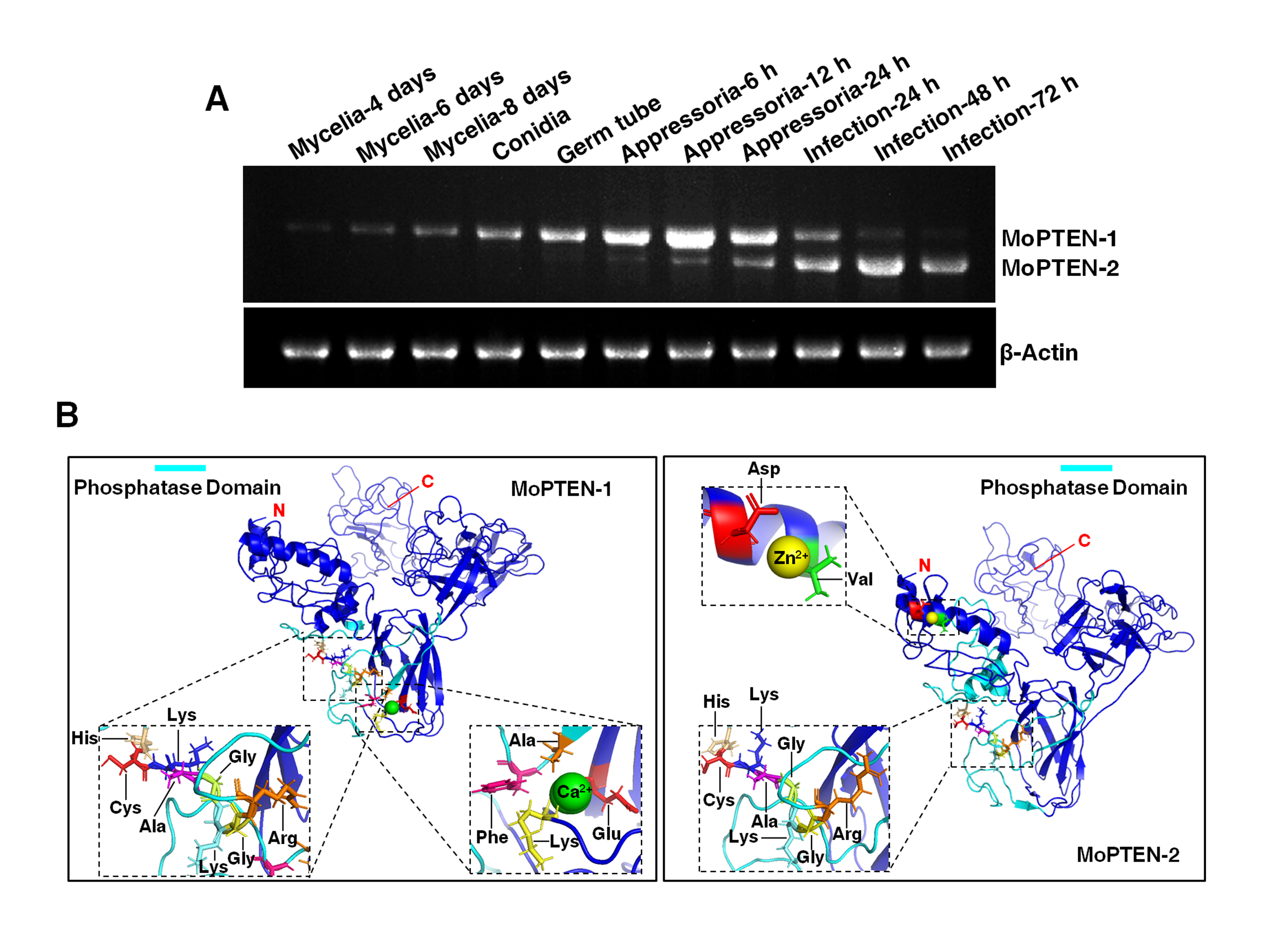

Supplement: Supplementary file 6 [file Image_5.TIF]

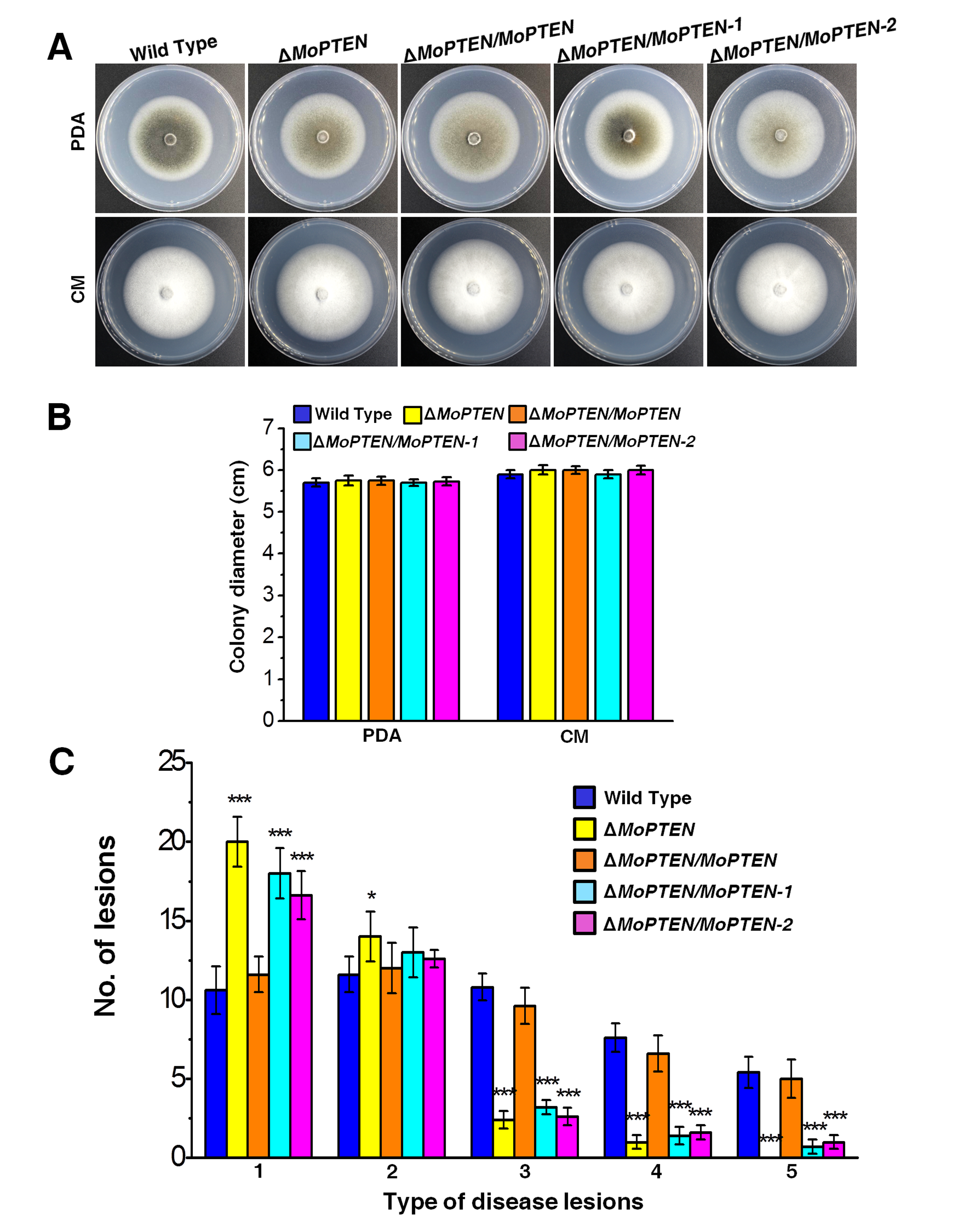

Supplement: Supplementary file 7 [file Image_6.TIF]

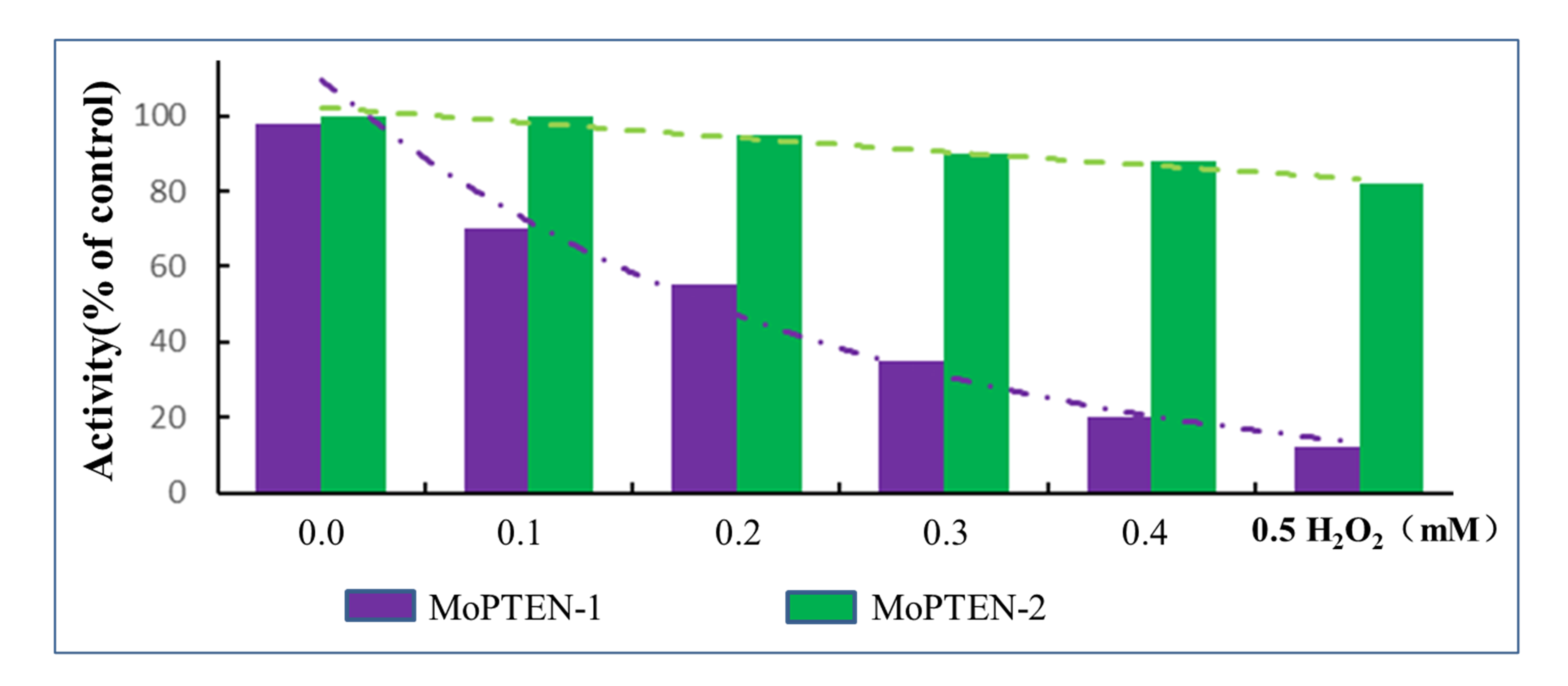

Supplement: Supplementary file 8 [file Image_7.TIF]

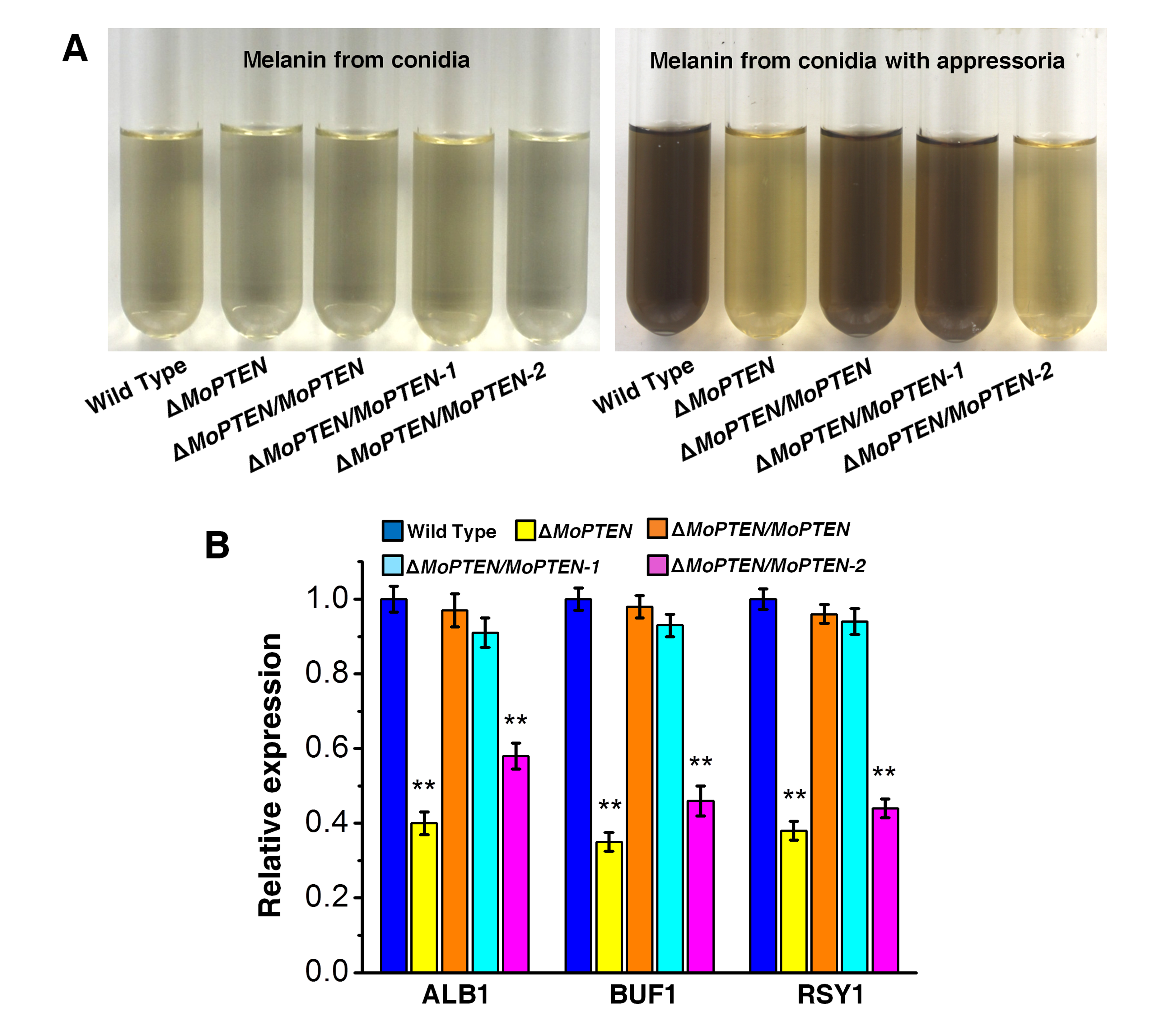

Supplement: Supplementary file 9 [file Image_8.TIF]

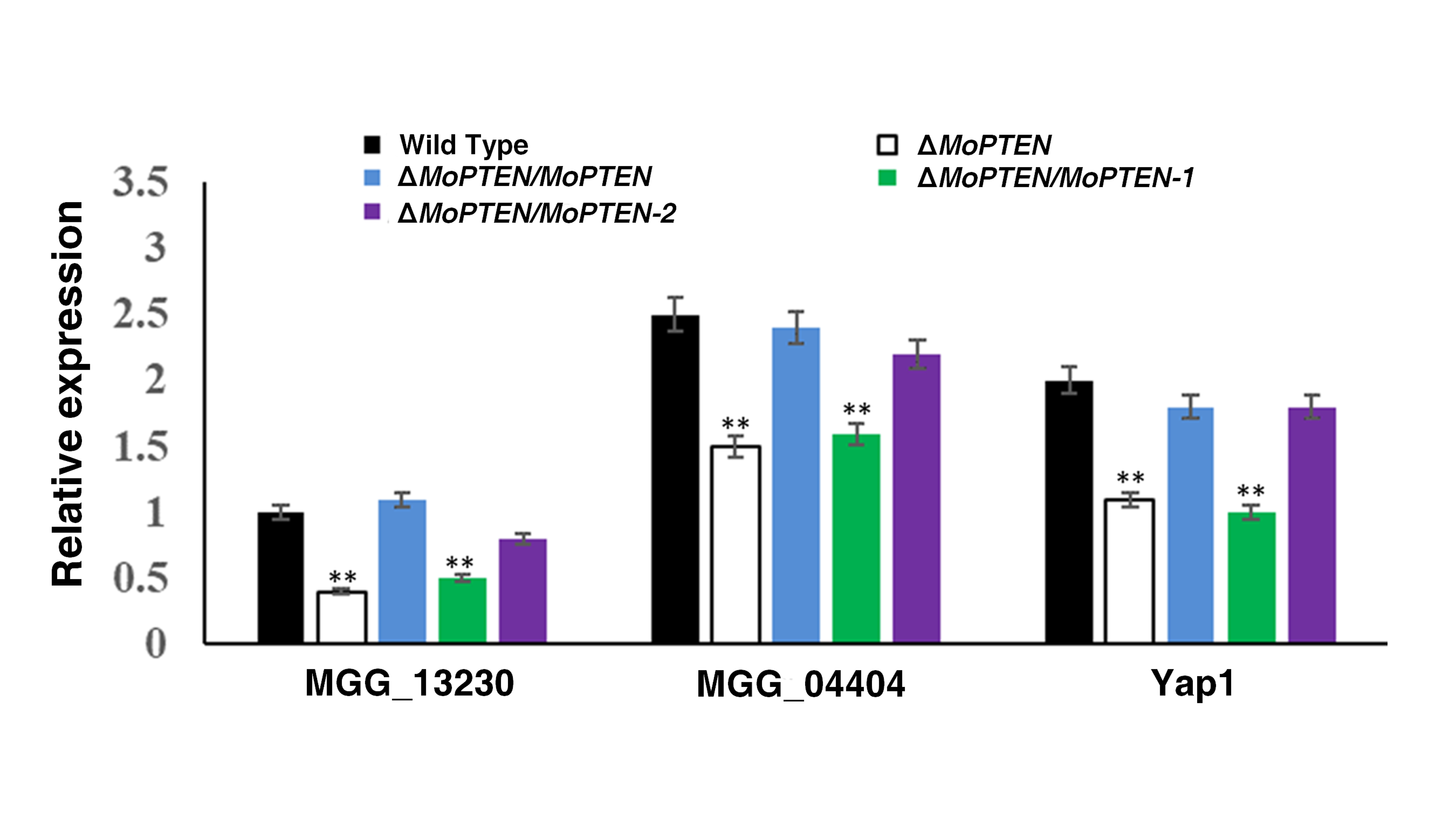

Supplement: Supplementary file 10 [file Image_9.TIF]
